# Anion Architecture Controls Structure and Electroresponsivity of Anhalogenous Ionic Liquids in a Sustainable Fluid

**DOI:** 10.1021/acs.jpcb.3c08189

**Published:** 2024-04-19

**Authors:** Sichao Li, Oliver S. Hammond, Andrew Nelson, Liliana de Campo, Michael Moir, Carl Recsei, Manishkumar R. Shimpi, Sergei Glavatskih, Georgia A. Pilkington, Anja-Verena Mudring, Mark W. Rutland

**Affiliations:** † Division of Surface and Corrosion Science, School of Engineering Sciences in Chemistry, Biotechnology and Health, KTH Royal Institute of Technology, Stockholm SE-100 44, Sweden; ‡ Department of Materials and Environmental Chemistry, Stockholm University, Stockholm SE-114 18, Sweden; § intelligent Advanced Materials, Department of Biological & Chemical Engineering and iNANO, Aarhus University, Aarhus C 8000, Denmark; ∥ Australian Centre for Neutron Scattering, ANSTO, Lucas Heights, New South Wales 2234, Australia; ⊥ National Deuteration Facility, ANSTO, Lucas Heights, New South Wales 2234, Australia; # Chemistry of Interfaces, Department of Civil and Environmental Engineering, Luleå University of Technology, Luleå SE-97187, Sweden; ∇ System and Component Design, Department of Engineering Design, KTH Royal Institute of Technology, Stockholm SE-100 44, Sweden; ○ School of Chemistry, University of New South Wales, Sydney, New South Wales 2052, Australia; ◆ Department of Electromechanical, Systems and Metal Engineering, Ghent University, Ghent B-9052, Belgium; ¶ Department of Physics, Umeå University, Umeå SE-901 87, Sweden; ▲ Bioeconomy and Health Department Materials and Surface Design, RISE Research Institutes of Sweden, Stockholm SE-114 28, Sweden; ▼ Laboratoire de Tribologie et Dynamique des Systèmes, École Centrale de Lyon, Ecully Cedex 69134, France

## Abstract

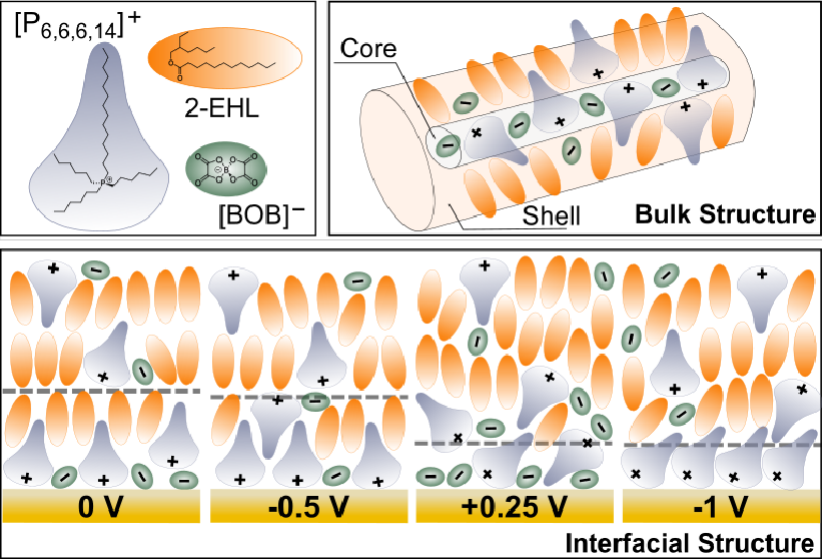

Three nonhalogenated ionic liquids (ILs) dissolved in 2-ethylhexyl laurate (2-EHL), a biodegradable oil, are investigated in terms of their bulk and electro-interfacial nanoscale structures using small-angle neutron scattering (SANS) and neutron reflectivity (NR). The ILs share the same trihexyl(tetradecyl)phosphonium ([P_6,6,6,14_]^+^) cation paired with different anions, bis(mandelato)borate ([BMB]^−^), bis(oxalato)borate ([BOB]^−^), and bis(salicylato)borate ([BScB]^−^). SANS shows a high aspect ratio tubular self-assembly structure characterized by an IL core of alternating cations and anions with a 2-EHL-rich shell or corona in the bulk, the geometry of which depends upon the anion structure and concentration. NR also reveals a solvent-rich interfacial corona layer. Their electro-responsive behavior, pertaining to the structuring and composition of the interfacial layers, is also influenced by the anion identity. [P_6,6,6,14_][BOB] exhibits distinct electroresponsiveness to applied potentials, suggesting an ion exchange behavior from cation-dominated to anion-rich. Conversely, [P_6,6,6,14_][BMB] and [P_6,6,6,14_][BScB] demonstrate minimal electroresponses across all studied potentials, related to their different dissociative and diffusive behavior. A mixed system is dominated by the least soluble IL but exhibits an increase in disorder. This work reveals the subtlety of anion architecture in tuning bulk and electro-interfacial properties, offering valuable molecular insights for deploying nonhalogenated ILs as additives in biodegradable lubricants and supercapacitors.

## Introduction

In recent years, the global challenges posed by climate change have heightened the urgency of replacing petrochemicals, enhancing energy efficiency, and fostering ecofriendly energy technologies. In pursuit of these sustainability objectives, the development of materials for energy storage, conversion, and the reduction of energy losses plays a pivotal role.^1,2^ Ionic liquids (ILs), tailored materials with unique and tunable physicochemical properties (e.g., low volatility and flammability, high thermal stability, a wide electrochemical stability window, and compatibility with organic compounds),^3,4^ have emerged as ideal candidates for the above applications.^5−9^

ILs are nominally molten salts with melting points below 100 °C.^10^ While they have been known for over a century,^11^ their transformative potential as solvents for advanced chemical technologies largely remained unnoticed until the past three decades. With the growing demand for electrification, ILs have experienced a resurgence in research interest across various applications, including supercapacitors, batteries, sensors, and lubrication.^12−14^ In the latter scenario, the electro-responsive properties of ILs enable the active control of advanced lubrication systems through electric fields, a concept known as “tribotronics”,^15^ making them promising materials for electric and hybrid electric vehicles.^16,17^ Nevertheless, several issues have impeded the commercialization of ILs, including high production costs and, in the current context, limited miscibility with conventional mineral oils.^18^ Furthermore, the prevalent use of halide-containing ILs (with the halide being part of one of the ions or as an impurity originating from the synthesis) poses a corrosion risk under harsh tribological conditions, characterized by gigapascal-range pressures and contact temperatures exceeding 100 °C, potentially leading to environmental hazards.^19−22^

To address these issues, a series of nonhalogenated ILs, primarily containing phosphorus- and boron-based ions, has been synthesized and developed.^18,23−26^ In addition to the absence of corrosive halides, boron-based ILs are well-regarded for their ability to reduce wear and friction through the formation of sacrificial tribofilms or lubricating boundary layers, while phosphonium-based ILs show enhanced thermal stability.^18,24,27^ These nonhalogenated ILs demonstrate promising antiwear and friction-reduction properties, both as neat lubricants^28−30^ and as additives.^31,32^ Prior efforts to characterize the properties and tribological performance of these nonhalogenated ILs have employed a range of experimental techniques, including atomic force microscopy (AFM),^33−35^ nuclear magnetic resonance (NMR),^36,37^ Fourier transform infrared (FTIR),^24,38^ scanning electron microscopy (SEM),^29^ time-of-flight-secondary ion mass spectrometry (ToF-SIMS),^28,39^ and macrotribology tests,^30,40^ as well as molecular dynamics (MD) simulations.^41,42^ For readers interested in the bulk properties of the studied ILs, we refer to a comprehensive thesis by Rohlmann.^43^ Recently, a macroscale tribological test system was developed that allowed studying the tribotronic control of phosphonium orthoborate ILs dispersed in 2-ethylhexyl laurate: 2-EHL, a biodegradable oil, revealing a systematic variation in lubricant film thickness when controlled by an applied electric field. Meanwhile, electrochemical neutron reflectometry (NR) was employed as a complementary probe to elucidate the nanoscale structural and compositional changes in the IL boundary films in response to different applied surface potentials, connecting the molecular control to the macroscopic tribotronic performance and controllability.^44^

In addition to NR, our constellation has conducted investigations of the bulk structure and interfacial electroresponsive behavior of various nonhalogenated ILs, both neat and as additives in base fluids (e.g., base oil, acetone, and propylene carbonate), using small-angle neutron scattering (SANS), quartz crystal microbalance (QCM), and sum frequency generation (SFG) spectroscopy, among other techniques.^45−51^ Recent SANS measurements, in particular, have provided direct evidence of bulk nanostructure in neat bis(orthoborate) ILs and how such structures and their intermolecular interactions are influenced by the specific choice of ion pairs and their molecular structures.^51^ Meanwhile, previous NR measurements have provided further information on the interfacial electroresponsive behavior and structural characteristics of nonhalogenated ILs and their mixtures with polar solvents at the liquid–solid interface under the influence of an external electric field.^47,48^ Recently, in conjunction with NR measurements, the assessment of surface charge via QCM has revealed a voltage-induced interphase transition phenomenon occurring in a phosphonium orthoborate-based IL.^46^

In this study, SANS and NR measurements have been conducted to examine the bulk structure and electro-responsive interfacial behavior of three phosphonium orthoborate-based ILs dispersed in the biodegradable oil 2-EHL. These ILs share the same trihexyl(tetradecyl)phosphonium ([P_6,6,6,14_]^+^) cation, while the orthoborate-based anions vary between bis(mandelato)borate ([BMB]^−^), bis(oxalato)borate ([BOB]^−^), and bis(salicylato)borate ([BScB]^−^). In comparison to other solvents used in previous studies, such as acetone and propylene carbonate,^46,47^ 2-EHL is recognized as a green ester-based biodegradable oil, finding practical utility in lubricant formulation due to its low viscosity and high spreading properties.^52−55^ Moreover, 2-EHL when fully deuterated possesses a high scattering length density (SLD), allowing optimized contrast for SANS and NR measurements. Together, these results establish a clear link between anion architecture and bulk structure as well as their interfacial electroresponsive behavior. In addition, a self-assembly interaction between the 2-EHL and the cation, both in the bulk and interface, is revealed and shown to be influenced by the specific nature of its paired anion.

## Materials and Methods

### Materials and Solution Preparation

**Figure 1:**
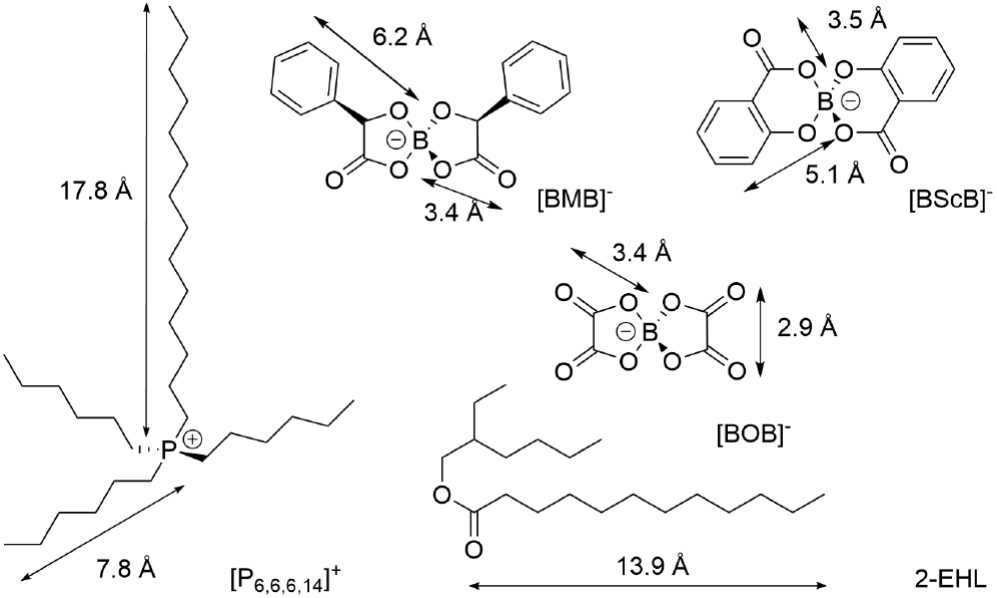
Molecular structures and dimensions of the IL ions and solvent, H-2-EHL. Dimensions were estimated using Avogadro software.^56,57^

Phosphonium orthoborate-based ILs trihexyl(tetradecyl)phosphonium-bis(mandelato)borate ([P_6,6,6,14_][BMB]), trihexyl(tetradecyl)phosphonium-bis(oxalato)borate ([P_6,6,6,14_][BOB]), and trihexyl(tetradecyl)phosphonium-bis(salicylato)borate ([P_6,6,6,14_][BScB]) were obtained from the Luleå University of Technology and utilized without additional purification.^24,25^ The structure and high purity of the ILs have been confirmed in previous publications through electrospray ionization mass spectrometry (ESI-MS) and multinuclear (^1^H, ^13^C, ^31^P, and^11^B) NMR spectroscopy. Water content was tested by coulometric Karl Fischer titration and was found to be 0.038 wt% for [P_6,6,6,14_][BMB], 0.06 wt% for [P_6,6,6,14_][BOB], and 0.033 wt% for [P_6,6,6,14_][BScB].^24,25^ The ILs were further dried under dynamic vacuum at 60 °C for approximately 72 h to eliminate volatile contaminants like atmospheric water prior to use on site. 2-Ethylhexyl laurate (H-2-EHL) was obtained commercially as Dehylub 4003 from Emery Oleochemicals GmbH. Perdeuterated 2-ethylhexyl laurate (D-2-EHL) was prepared according to a previously described procedure by the National Deuteration Facility of the Australian Nuclear Science and Technology Organisation (ANSTO).^55^ Both solvents, H/D-2-EHL, were used without further treatment. Figure 1 illustrates the molecular structure and dimensions of the ILs and 2-EHL.

For solution preparation, the respective IL was weighed and proper amounts of deuterated and hydrogenated 2-EHL were added to achieve contrast-matching requirements. For the SANS measurements, pure D-2-EHL was selected as the solvent to maximize the SLD contrast with the IL ions. For the NR measurements, to maximize the measurement sensitivity for detecting any changes in the interfacial region, the SLD of the bulk IL/2-EHL solutions was contrast-matched with that of gold by an appropriate combination of H-2-EHL and D-2-EHL. Table 1 provides data on the density, molecular volume, and SLD of both ions and solvent. The ion density and molecular volume were estimated through atomistic simulations.^45^ The theoretical SLDs of ILs were calculated based on their measured mass density using an Anton Paar density meter (DMA 4500 M).

**Table 1:** Densities, Molecular Volumes, and SLDs of IL Ions and Solvent

**species**	**ρat 25 °C(g/cm**^**3**^**)**	***V*_*M*_(Å**^**3**^**)**	**SLD(**× **10**^**–**6^**Å**^**–2**^**)**
[P_6,6,6,14_][BMB]	1.023	1291	0.50
[P_6,6,6,14_][BOB]	0.997	1118	0.37
[P_6,6,6,14_][BScB]	1.019	1247	0.53
[P_6,6,6,14_]^+^	0.880	914	–0.40
[BMB]^−^	1.376	375	2.71
[BOB]^−^	1.521	204	3.84
[BScB]^−^	1.411	333	3.10
H-2-EHL	0.860	604	–0.08
D-2-EHL	0.971	602	6.70

Each solution was thoroughly mixed using either a vortex mixer or an ultrasonicator (maximum 10 min) until homogeneity (by visual assessment) and was promptly utilized. The stability and water content of the IL solutions were assessed using Fourier transform infrared spectroscopy (Nicolet iS 10 FT-IR), both before and after the NR measurement. Infrared spectra of IL/2-EHL solutions employed in the NR measurements are presented in the Supporting Information and exhibited no discernible water peaks.

### Small-Angle Neutron Scattering (SANS)

Small-angle neutron scattering (SANS) measurements were conducted on the BILBY SANS instrument located at ANSTO, Sydney, Australia. Samples were measured in constant-wavelength mode, using a velocity selector to achieve an incident neutron wavelength of 4.5 Å (), where *Q*, the momentum transfer vector, is defined as *Q* = (4π sin θ)/λ, with 2θ being the scattering angle and λ the wavelength of the incident neutron beam). An optimized arrangement of rear and “curtain” detectors gave an accessible *Q*-range of 0.0036 ≤ *Q* ≤ 0.7212 Å^–1^ (i.e., corresponding to real-space distances of 9–1750 Å, with a maximum formal structural resolution of 4.4 Å). Measurements were made of the direct beam, blocked beam, and standards including an empty 1-mm quartz Hellma cell, to allow reduction of the raw neutron scattering data to absolute units using Mantid.^58^ It should be noted that some very slow equilibration times have been observed in ILs using scattering techniques.^59^ Such behavior was not observed for these dilute systems.

Following subtraction of the solvent background, scattering profiles were analyzed using a model-based fitting approach implemented in SasView.^60^ Best fits to the data were obtained using a model for high aspect ratio cylindrical aggregates, with a diffuse corona (or “shell”). The SLD (**ρ**) of the “core” was fixed to the values for the neat ILs given in Table 1 (note that the fitting is rather insensitive to this parameter), and the SLD of the bulk solvent (D-2-EHL) was 6.7 **×** 10^–6^ Å^–2^. The incoherent background *c*_0_, scaling factor, cylinder length, radius, shell diameter, and shell SLD were fitted as the free parameters. No interaction potential, i.e., structure factor *S*(*Q*), was included, since it was not found to be necessary to achieve the best quality of fit.

### Neutron Reflectivity (NR)

#### Electrochemical NR Cell and Gold Electrode Preparation

Neutron reflectivity (NR) measurements of IL/2-EHL solutions were conducted using a custom-made electrochemical NR cell, the design of which has been previously described in studies involving ILs in acetone and propylene carbonate systems.^46−48^ A thin, amorphous gold film (approximately 160 Å) served as the working electrode (WE) and was coated onto a 10-mm-thick (50 × 50 mm, *w* × *l*) polished silicon (100) block (Sil’tronix Silicon Technologies). An adhesion layer, either titanium (in 5% w/w [P_6,6,6,14_][BxB] in 2-EHL) or chromium (in 2.5% w/w [P_6,6,6,14_][BMB] and 2.5% w/w [P_6,6,6,14_][BOB] Mixture in 2-EHL), was predeposited on the silicon surface. A short, insulated copper wire was affixed to a corner of the gold surface using conductive epoxy CW 2400 (Chemtronics) and then cured at 120 °C in an oven for 20–30 min. Subsequently, the gold surface was rinsed with filtered absolute ethanol, dried in a stream of nitrogen gas, and exposed to UV/ozone for 10 min to eliminate any organic residues.

For the presented measurements, a two-electrode system was employed due to space constraints in the NR electrochemical cell configuration. The counter/reference electrode (CE/RE) was constructed using a conductive glass coated with fluorine-doped tin oxide (FTO, Sigma Aldrich). To separate the CE/RE from the WE and contain the IL/2-EHL solution, a 0.5-mm-thick PTFE gasket was utilized. Prior to assembling, all cell components, including the conductive glass and gasket, underwent a 30-min ultrasonic treatment in 2% v/v Hellmanex at room temperature. Subsequently, they were rinsed thoroughly with Milli-Q water and filtered with absolute ethanol before nitrogen drying. Cell assembly occurred within a polyethylene glovebag in a dry argon atmosphere (relative humidity < 10%) to minimize ambient water contamination. The prepared IL/2-EHL solutions were introduced into the cell via an adaptor and a glass Luer syringe.

After the cell was mounted on the beamline, an Autolab PGSTAT204 potentiostat (Metrohm) was connected to electrodes for applying potentials across the cell. The applied potentials, which were selected within the stable electrochemical window of the ILs, determined from cyclic voltammetry (CV) in prior studies,^46,48,61^ were applied in the same sequence as presented. Continuous monitoring and recording of the applied potentials and current across the cell were conducted throughout the NR measurements. Before each NR measurement, the cell was allowed to stabilize at the applied potential for 30 min to reach equilibrium. The surface charge density was estimated based on the effective electrode surface area (defined by PTFE gasket, 42 × 42 mm^2^) and on the change in current during the equilibrium time, as illustrated in Figure S4. Following the completion of NR measurements, CV was conducted across each IL/2-EHL solution in the cell to assess the occurrence of any Faradaic events during the NR experiment and evaluate the reversibility of charge transfer within the studied potential range.^47^ The post-NR CV measurements for all systems are available in the Supporting Information, and there is no evidence of any oxidative or reductive processes/reactions.

#### NR Measurements

Specular neutron reflectivity (*R*), the ratio of intensity between the reflected and the incident beam, was determined experimentally by varying the momentum transfer vector (*Q*). The experimental setup was involved in directing the neutron beam through the silicon block and reflecting it at the gold–solution interface back through the silicon block toward a detector.

The NR measurements in this study for the solutions 5% w/w [P_6,6,6,14_][BMB], [P_6,6,6,14_][BOB], and [P_6,6,6,14_][BScB] in 2-EHL (5% w/w [P_6,6,6,14_][BxB] in 2-EHL) and 20% w/w [P_6,6,6,14_][BMB] and [P_6,6,6,14_][BOB] in 2-EHL (presented in Figure S10) were performed using the PLATYPUS time-of-flight neutron reflectometer at ANSTO in Sydney, Australia.^62^ To cover the *Q* range from the critical edge to the background (0.008–0.22 Å^–1^), two incident angles, 0.65° and 3°, were employed. A constant Δ*Q*/*Q* resolution of approximately 5% was maintained across all the measurements. To ensure a consistent beam footprint within the region covered by the gold–solution interface, slits were employed to precisely define the beam size. The NR measurements were performed with the electrochemical NR cell placed in a horizontal configuration, with the exception of the NR measurements for the 2.5% w/w [P_6,6,6,14_][BMB] and 2.5% w/w [P_6,6,6,14_][BOB] mixture in 2-EHL (see the 2.5% w/w [P_6,6,6,14_][BMB] and 2.5% w/w [P_6,6,6,14_][BOB] Mixture in 2-EHL section), which were conducted vertically on the SuperADAM reflectometer at the Institut Laue-Langevin (ILL), Grenoble, France.^63,64^ For the mixture system experiments, the wavelength remained constant at 5.21 Å, while the angle of incidence was adjusted, resulting in a *Q* range of 0.005–0.21 Å^–1^ with a resolution of Δλ/λ = 0.005. Standard data reduction procedures were applied, encompassing background subtraction, detector efficiency calibration, overillumination correction (for data collected from SuperADAM), and subsequent normalization to reflectivity based on the direct beam. For PLATYPUS, data sets from the two incident angles were combined to generate a unified NR profile. The reflectivity data error bars presented are computed from a Poisson distribution, where the count error is equal to the square root (or standard deviation) of the number of counts measured at each *Q* position.

#### NR Data Analysis

The reduced data were fitted using the GenX software package^65^ within a limited *Q* range (0.008–0.13 Å^–1^) due to the significant background noise and lack of Kiessig fringes beyond *Q* = 0.13 Å^–1^. SLD profiles were obtained through best fits to the NR profiles based on the lowest figure of merit (FOM) values. Fitting employed a slab model comprising a series of stratified layers with varying thickness, roughness, and SLD. Given the comparable roughness of gold (∼10 Å) to the dimensions of the IL ions (cf. Figure 1), a “micro-slabbing” approach (also known as “slicing”),^66,67^ which has been previously implemented for other IL–solvent systems,^46,49^ was employed for the *gold–innermost layer* interfacial region.

In brief, this approach subdivided the *gold–innermost layer* interface into a sequence of 1-Å-thick slabs of 0 Å roughness. The SLD of each microslab was determined based on the normal distribution of the volume fraction of gold and innermost layer to describe a smooth transition. Rather than fitting the roughness of the gold interface, this approach instead describes the thickness of this interfacial region (*t*_i_). The cumulative distribution function in SciPy^68^ was utilized to compute the normal distribution, with the scale set to 3*t*_i_/20 to ensure that the volume fraction approached 0 and 1 at the edges of *t*_i_.^49^ This thickness of the interfacial region, along with Au thickness and bulk solution SLD, was fitted and fixed after the first applied potential (i.e., 0 V in this study). All other parameters related to the substrate (i.e., SLD, thickness, and roughness of the Ti and SiO_2_ layer, as well as roughness of the Si layer, see Supporting Information) were determined from the NR measurements of the block in air and remained constant for applied potentials. It is noteworthy that, due to experimental time constraints, the substrate parameters for the 2.5% w/w [P_6,6,6,14_][BMB] and 2.5% w/w [P_6,6,6,14_][BOB] mixture in 2-EHL (see the 2.5% w/w [P_6,6,6,14_][BMB] and 2.5% w/w [P_6,6,6,14_][BOB] Mixture in 2-EHL section) were directly fitted and fixed after the first applied potential, using a block from the same deposition batch (and characterized in our previous study)^46^ and as a starting reference.

The thickness, roughness, and SLD values of the interfacial IL–solvent layers were allowed to vary for all fitted data sets. Initially, a one-layer slab model was applied, and additional layers were introduced to the fit if the FOM value was found to significantly improve (i.e., decrease). No predefined interfacial structures were assumed. Typically, the roughness of layers is commonly limited to not more than 30% of their layer thickness. The SLDs of the layers were constrained within the range of possible component species.

## Results and Discussion

### Small-Angle Neutron Scattering

**Figure 2:**
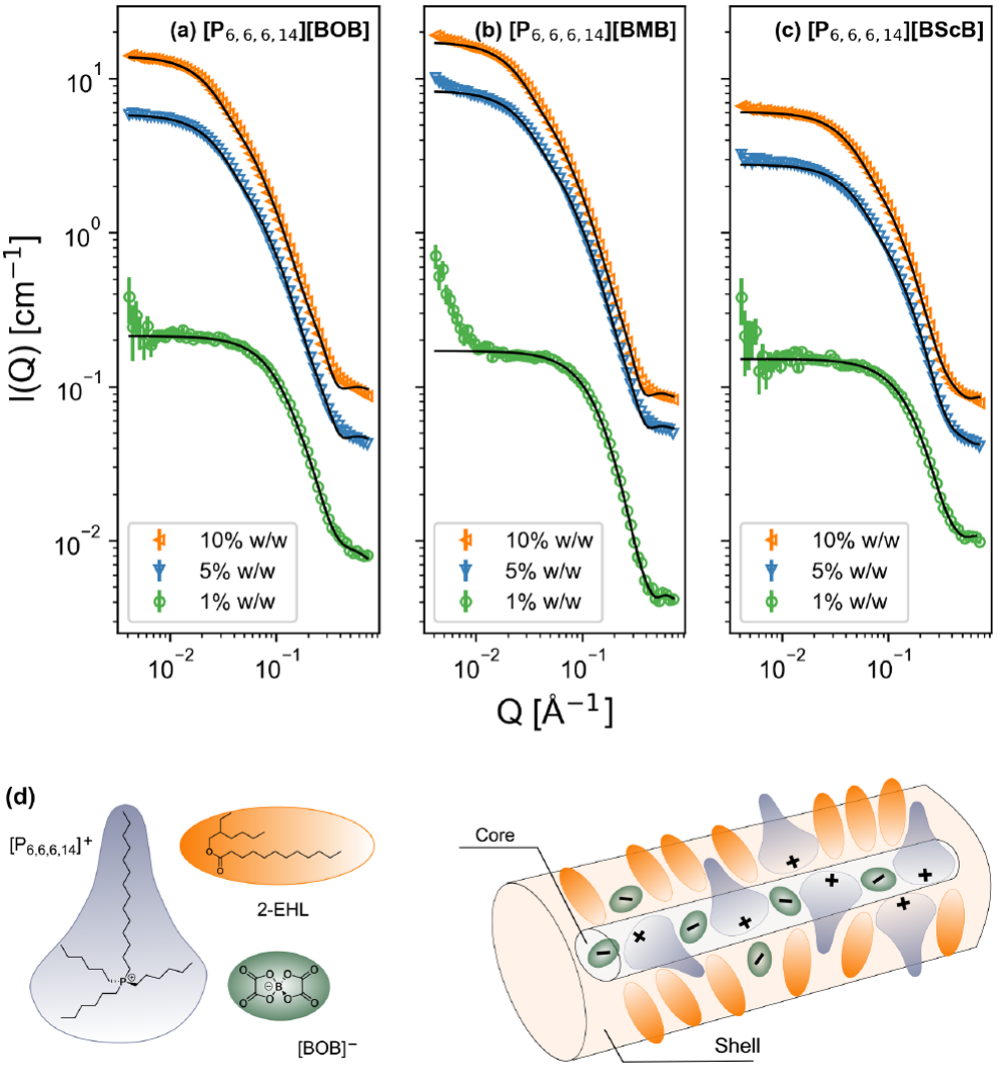
(a)–(c) SANS data (markers) measured for different concentrations of [P_6,6,6,14_][BxB]/D-2-EHL mixtures (x = O, M, Sc): 1% w/w IL (green circles), 5% w/w (blue downward triangles), and 10% w/w (orange sideways triangles). The solid (black) lines are fits to the data using a core–shell cylinder model; (d) cartoon representation of the local structuring in the core–shell cylinder model, comprising a central core of ion-paired [P_6,6,6,14_]^+^ cations and [BOB]^−^ anions, surrounded by a diffuse solvent-rich corona with sparsely solvated alkyl chains.

SANS measurements were performed to determine if the ILs form self-assembled structures in the bulk when dispersed at different concentrations in 2-EHL. Fluctuations in *I*(*Q*) were observed down to very low values of *Q*, demonstrating the existence of large-scale structures (see the data and fits in Figure 2). Numerous form factor models were tested and compared, including spheres and ellipsoids. However, best fits to all data sets were achieved with a core–shell cylinder model, consisting of a diffuse shell or corona, where the SLD of the shell was fitted as a free parameter (see the Materials and Methods section for more details). As shown in Table 2, the SLD value for the aggregate solvation corona is slightly below that of the pure D-2-EHL solvent (cf. Table 1). The model thus assumes that the core region comprises anions and the charged portion of the phosphonium cation, and the SLD for the respective neat IL was used for this region of ion pairs and oligomers. The shell is consequently a solvent-rich region of ca. 8–18 Å around the aggregate, which contains extended alkyl tails of the phosphonium cations (cf. Figure 2d). Interactions between alkyl chains, possibly including intercalation, seem the most likely reason for a solvation shell, given the nature of the species, although formally, based on the SLDs alone, the presence of anions in the shell cannot be ruled out. The width of this shell region varies markedly depending on the anion; for [BScB]^−^, it never exceeds 10 Å, whereas for [BOB]^−^ and [BMB]^−^, the shell reaches almost 20 Å. If the shell diameter is used as an indirect probe for how tightly bound the anions are, then the results suggest that [BOB]^−^ has more opportunity to rearrange than [BMB]^−^, and [BScB]^−^ is most tightly bound to the aggregate in the EHL base oil. Such observations are in alignment with the formation of a homogeneous one-liquid-phase IL matrix for pure [P_6,6,6,14_][BScB] demonstrated by a recent NMR diffusion study^36^ and prior observations that observed 1.5% dissociation of [P_6,6,6,14_][BOB] in (H-)2-EHL, compared to 0.3% for [P_6,6,6,14_][BMB].^53^

**Table 2:** Fit Parameters Obtained from the Core–Shell Cylinder Model Were Compared to the SANS Data, Including Cylinder Length, Core Radius (*R*_core_), Shell Diameter, Shell SLD, and Aspect Ratio (AR)

**IL**	**IL(% w/w)**	***L*[Å]**	***R*_core_[Å]**	**Shell[Å]**	ρ**_shell_(× 10**^**–6**^**Å**^**–2**^**)**	**AR**
[P_6,6,6,14_][BOB]	1	35.7 ± 1.5	4.1 ± 1.0	8.4 ± 0.8	6.13 ± 0.20	4.4
	5	150.8 ± 0.5	8.6 ± 0.0	16.5 ± 0.1	6.47 ± 0.00	8.8
	10	172.6 ± 0.4	9.0 ± 0.0	18.7 ± 0.0	6.49 ± 0.00	9.6
[P_6,6,6,14_][BMB]	1	24.8 ± 2.5	8.3 ± 0.2	12.1 ± 0.7	6.54 ± 0.04	1.5
	5	148.0 ± 0.4	8.8 ± 0.0	15.4 ± 0.1	6.47 ± 0.00	8.4
	10	170.2 ± 0.4	9.2 ± 0.0	17.9 ± 0.1	6.49 ± 0.00	9.3
[P_6,6,6,14_][BScB]	1	23.2 ± 2.1	7.6 ± 0.2	10.7 ± 1.2	6.60 ± 0.05	1.5
	5	82.5 ± 0.3	5.5 ± 0.1	8.0 ± 0.1	6.02 ± 0.01	7.4
	10	92.9 ± 0.2	6.6 ± 0.0	9.3 ± 0.0	6.22 ± 0.01	7.0

For all the ILs studied, the geometry of the observed self-assembly structures in the bulk strongly depended upon the sample composition, with both structural (i.e., anion identity) and concentration effects. In particular, the aggregate size expanded as a function of increasing concentration. Although the core radius (*R*_core_) increases somewhat with the IL concentration, the length increase is more significant, meaning that higher concentrations yield more highly extended aggregates with greater aspect ratios (ARs), up to lengths of ca. 170 Å for [BOB]^−^ and [BMB]^−^ samples at 10% w/w. The overall “tube” dimensions are commensurate with interferometric measurements of confined films under tribological conditions (ca. 60–180 Å).^44^ Structures with similar AR, such as worm-like micelles, have been studied extensively due to their ability to modify the overall physical properties, in addition to inducing emergent behaviors such as non-Newtonian shear response.^69^ When considering the molecular dimensions involved, our model therefore suggests the following: at 1% w/w, the dimensions of the well-defined cylindrical core region correspond to those of the known dimensions of individual ion pairs and dimers; at the higher concentration, the radius remains less than the size of an ion pair, but the length is much greater, indicating a growth into oligomeric ion chains with elements resembling traditional inverse micelles at 5% w/w and 10% w/w (Figure 2d). [BScB]^−^ with its larger orthoborate ring structure is generally more insensitive to concentration, displaying significantly shorter lengths (almost a factor of 2) and a denser core region between 5 and 10% w/w, which may be related to the strength of ion binding and/or degree of dissociation as discussed above. The core–shell model complicates the accurate calculation of the scaling factor, which should be closely related to the volume fraction of the scatterer (i.e., assembled IL). Since the aggregate structures appear to contain a rather large volume of solvent, this variable is fitted: in the example of the smaller [BScB]^−^ aggregates, if the total volume fraction of assembled IL remains identical across samples, then the *number fraction* of smaller aggregates must be higher (cf. Table S1), but the [BScB]^−^ aggregates display a smaller shell, which reduces the apparent volume fraction, even though the mole fraction of IL may be identical.

It is striking that the aspect ratio is so high. The combination of the known ion dimensions and the fitted form factor implies a “linear” self-assembly of either alternating ions or ion pairs, guided by Coulombic and/or solvophobic interactions: this interpretation allows for charge-balanced aggregates of the correct size, which are likely considering the low dielectric of the dispersant oil and the low dissociation of the ILs. Such linear assemblies, driven by local attractive energy minima and stabilized by a common corona, have been observed in nanoparticle systems^70^ and are relatively uncommon for molecular self-assembly systems, in part due to the lower propensity for self-assembly in media with low values of dielectric constant and cohesive energy density, cf. the Gordon parameter.^71,72^ These SANS results therefore offer a framework for understanding nanoscale structuring in these systems, which appears to be Coulombically dominated in the bulk, as has been suggested for other systems.^36,51^

### Neutron Reflectivity

#### 5% w/w [P_6,6,6,14_][BxB] in 2-EHL

As described in the Materials and Methods section, the bulk SLD of the IL/2-EHL solutions matched that of gold to maximize the sensitivity to any alterations in the near-surface interfacial region. Given the distinct SLDs of the component ions and solvent molecules, variation in SLD resulting from ion exchange at the electrified surface offers insights into both the composition changes and interfacial layer thickness.^46,47^

The reflectivity curves for 5% w/w [P_6,6,6,14_][BMB], [P_6,6,6,14_][BOB], and [P_6,6,6,14_][BScB] in 2-EHL solutions at a gold electrode surface at different applied potentials are shown in Figure S7. For all the different IL and potential conditions, NR shows Kiessig fringes, indicating substantial SLD contrast between the interfacial region and the gold electrode. The absence of interfacial structuring of the IL (or the solvent) at the gold interface would result in a featureless NR curve (dashed line in Figure S7). With applied potentials, the shift in location and amplitude of the fringe oscillations indicates the change of interfacial layer thickness and SLDs, respectively. For all three ILs studied here, under bias voltages, reflectivity differences (with respect to 0 V) reveal pronounced oscillatory behavior, reflecting electro-induced changes (i.e., both thickness and SLD changes of interfacial layers) at the electrified gold interface (cf. Figure S7 inset). Additionally, the NR data, plotted on the Fresnel representation scale RQ^4^, are shown in Figure S8 to highlight these changes.

**Figure 3:**
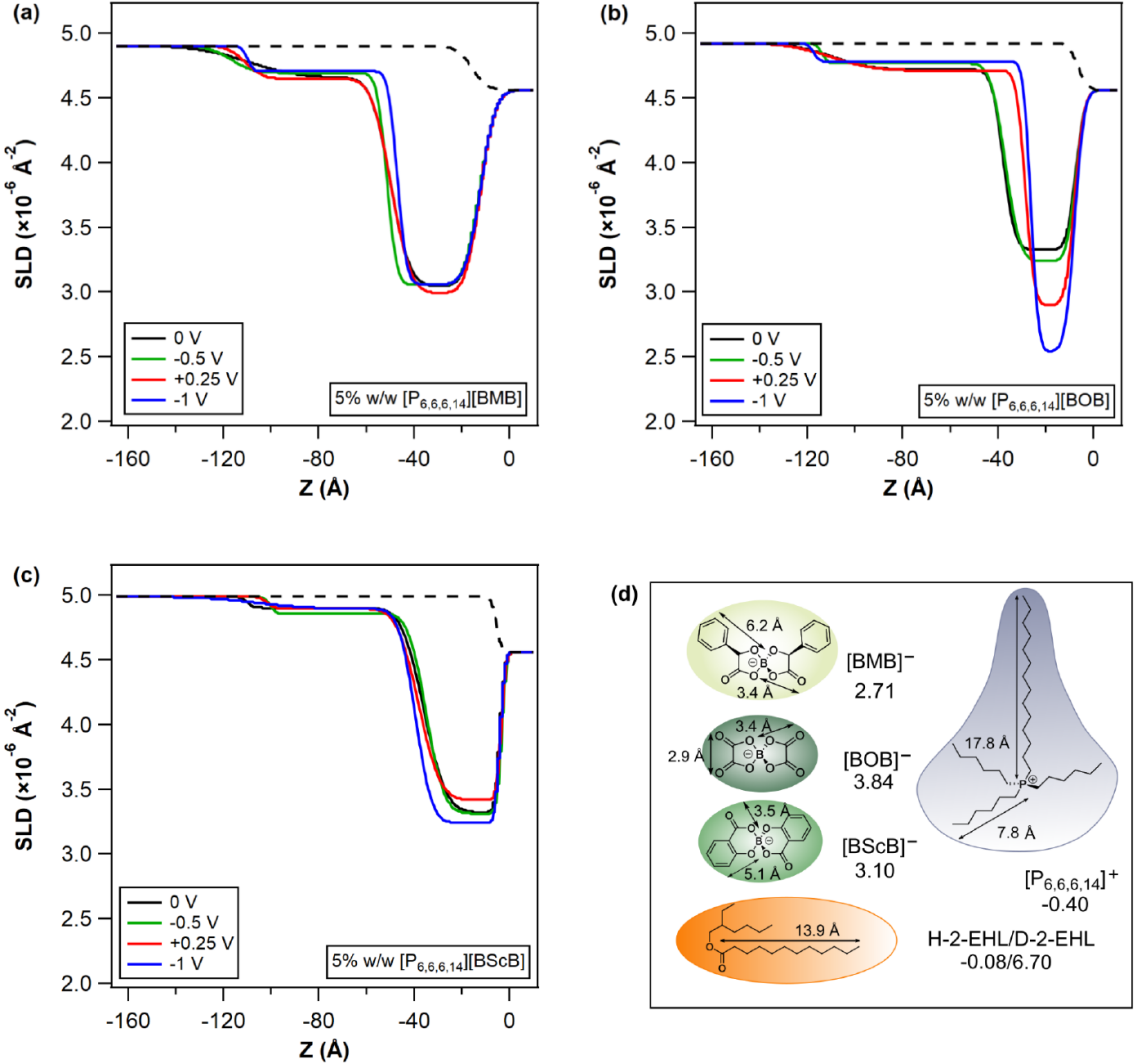
SLD profiles for 5% w/w (a) [P_6,6,6,14_][BMB], (b) [P_6,6,6,14_][BOB], and (c) [P_6,6,6,14_][BScB] in 2-EHL solutions, obtained from the best fits to the NR measurements at different applied potentials (cf. Figure S7) as a function of distance *Z*, where the gold–solution interface is located at *Z* = 0. The potential applied order is indicated by the legend. The dashed lines represent the SLD profile for the same gold surface and bulk IL/2-EHL solution that would be *predicted* in the absence of an adsorbed structured layer. (d) The SLD values (×10^–6^ Å^–2^) and dimensions for the different molecular constituents of the solutions.

Figure 3 displays SLD profiles obtained from the best fits to reflectivity curves for 5% w/w [P_6,6,6,14_][BxB] in 2-EHL solutions (solid lines in Figure S7) at different applied potentials. The fitted layer parameters (thickness, roughness, and SLD values) corresponding to these profiles are listed in Tables S4–6. A two-layer slab model consistently yielded best fits for all the ILs and potential conditions studied, supported by the lowest FOM values (refer to Figure S9). These models depict an *ion-rich* innermost layer with a compact structure, followed by a diffuse, *solvent-rich* (i.e., 2-EHL rich) outer layer with an SLD value close to that of the bulk, which aligns well with the observed solvent-rich shell from the above SANS measurements (cf. the Small-Angle Neutron Scattering section under Results and Discussion the section).

Previously reported NR measurements have examined the interfacial structuring of [P_6,6,6,14_][BMB] and [P_6,6,6,14_][BOB] in propylene carbonate (PC) at a charged gold interface at similar bulk IL concentrations.^46,48^ In that more polar solvent system, only IL-enriched interfacial layers were observed, even when multiple layer models were examined. The presence of the solvent-rich outer layer in IL/2-EHL systems studied here can be attributed to the structural characteristics of 2-EHL. Unlike PC, which is a cyclic carbonate ester, 2-EHL features a configuration where the polar ester linkage is partially separated from the apolar alkyl tails, oriented parallel and away from the oxygen-rich “head group”.^53,55^ This “surfactant-like” structure introduces some degree of self-assembly, or interpenetration, with the [P_6,6,6,14_]^+^ cation layer through intermolecular interactions with its long alkyl chain, resulting in the formation of the diffuse solvent-rich (secondary) layer. In contrast, the distinct electro-responsive behavior of the ion-rich innermost layer seen in these systems depends on the nature of the specific anion, which will be discussed in more detail below.

SLD profiles of 5% (w/w) [P_6,6,6,14_][BMB] in 2-EHL (cf. Figure 3a) point toward [BMB]^−^ being the least electroresponsive anion due to its limited dissociation.^53^ This weak responsivity aligns with previous findings regarding [P_6,6,6,14_][BMB] on gold in other polar solvents.^47,48^ In addition, ^11^B NMR measurements reported that the [BMB]^−^ anion exhibits a lower diffusion coefficient than [BOB]^−^ and [BScB]^−^ anions.^36^ At −1 V, a small but unambiguous change in the innermost layer thickness indicates a consolidation of [P_6,6,6,14_]^+^ cations at the gold interface to compensate the larger surface charge. Compared to the low electroresponse of the innermost layer, the outer solvent-rich layer exhibits a relatively pronounced response under different bias potentials, despite it consisting predominantly of solvent (cf. Figure 3a and Table S4 second layer). At −0.5 and −1 V, the thicker outer layer with a higher SLD suggests the formation of a dispersed structure containing more 2-EHL due to the increased proximity of the cation polar group close to the surface and the ensuing tendency of the longer alkyl tails to point toward the bulk. There may also be an influence of an ion exchange process either of counterion ([P_6,6,6,14_]^+^ cation) moving from the bulk or coion ([BMB]^−^ anion) being expelled from the innermost layer.

5% w/w [P_6,6,6,14_][BOB] in 2-EHL displayed the most significant electroresponse, which is consistent with its behavior in the more polar solvent, PC, where a transition from a bilayer structure to a conventional electric double-layer (EDL) configuration was observed.^46^ This substantial response can be attributed to the greater ability of the ions to move independently, observed earlier through the higher ion dissociation^53^ and diffusion^36^ of the [BOB]^−^ anion. This in turn leads to a significant potential-induced rearrangement from a random adsorbed film to an electrically conditioned checkerboard structure. Note that the NR data for 0, +0.25, and −1 V in Figure S7b were recently reported together with elastohydrodynamic (EHD) lubrication tests, where they supported arguments for thickness changes in films in a rolling contact with applied potential. That study demonstrated a marked electroresponse of the lubricant film thickness in the 5% w/w [P_6,6,6,14_][BOB]/2-EHL system with external electric fields.^44^ The SLD profiles presented herein are based on the same raw data but with a refined fitting procedure and additional data for −0.5 V included. The electro-responsive NR behavior could not be *exactly* matched with tribological test outcomes due to differences in the substrate (a steel substrate was used for the EHD macro-tribological tests), but the boundary layer thickness determined by NR (∼140 Å, as shown in Figure 3b) is in good agreement with the initial lubrication film thickness of the same system at 0 V. Furthermore, upon application of a negative potential (−1 V), a thinning of the layer was similarly observed in the rolling contact film thickness, which was reaffirmed by the electro-responsivity of well-defined boundary layers in NR measurements.^44^

**Figure 4:**
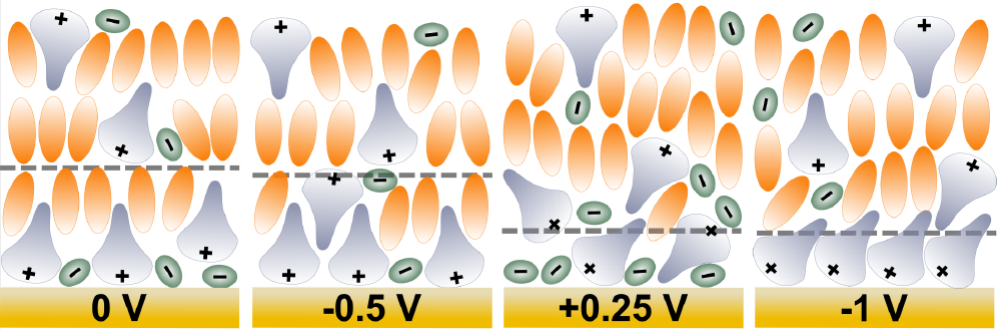
Schematics showing structure and composition change of interfacial layers under neutral and polarized potentials for 5% w/w [P_6,6,6,14_][BOB] in 2-EHL solution. The innermost layer is ion-rich, and the outer layer is solvent-rich. The schematics from left to right are arranged according to the applied voltage order.

To elaborate further, at −0.5 V, the slightly reduced SLD of the innermost layer compared to 0 V suggests a higher proportion of [P_6,6,6,14_]^+^ cations. At +0.25 V, the innermost layer thickness is approximately halved, indicative of an electro-conditioning from a “solvent”-rich *innermost* layer containing both cations (probably with multiple cation orientations) and anions, to a thinner ion-rich interfacial layer (cf. Figure 4). This (anion-rich) layer is essentially a more ordered checkerboard structure with less solvent content, reducing the SLD of the innermost layer. The increased outer layer thickness with a lower SLD compared to previously applied potentials (i.e., 0 and −0.5 V) indicates an accumulation of expelled ions (and possibly increased Coulombic ordering) in the solvent-rich layer. This secondary layer also implies a continued cation presence in the innermost layer for intercalation interactions, as does the relatively low SLD of the first layer. This electro-conditioned checkerboard (or structural transition of the adlayer) appears irreversible, as suggested by the consistently low innermost layer thickness and SLD at the subsequent −1 V potential. Related hysteretic interfacial ordering has been observed for this IL in PC; there a clear interphase transition occurs,^46^ as opposed to the electro-conditioning behavior observed here.

5% w/w [P_6,6,6,14_][BScB] in 2-EHL presents limited electroresponse under applied potentials (cf. Figure 3c), which once again correlates with the strong ion binding suggested by the IL aggregate structures observed in the bulk SANS profiles (see the Small-Angle Neutron Scattering section under the Results and Discussion section). In 2-EHL, this greater association could contribute to a somewhat reduced self-assembly interaction between the [P_6,6,6,14_]^+^ cations and 2-EHL molecules, resulting in an outer solvent-rich layer with the SLD more closely matching that of the bulk solution. In contrast to the weak innermost layer response for [P_6,6,6,14_] [BMB], 5% w/w [P_6,6,6,14_][BScB] in 2-EHL displays a measurable change in the innermost ion-rich layer. For +0.25 V, the increase in SLD and thickness of the innermost layer can be attributed to the attraction of the [BScB]^−^ anion (or ion pair due to the strong ion binding) to the positively electrified surface. This situation is mirrored at large negative potentials (i.e., −1 V), where the reduction in SLD and increase in the thickness of the innermost layer are associated with the attraction of [P_6,6,6,14_]^+^ cations, also arising from [P_6,6,6,14_][BScB] ion pairing. Furthermore, in comparison to the neutral potential (0 V), the decreased thickness of the outer solvent-rich layer at polarized potentials suggests a more compact arrangement of counterions to compensate the excess surface charge (see Table S6). The behavior of the [BScB]^−^ system is thus intermediate between that of [BOB]^−^ and [BMB]^−^, but more similar to that of [BMB]^−^.

As a result, only [P_6,6,6,14_][BOB] and [P_6,6,6,14_][BMB] as representatives of the extremes of electroresponsiveness were selected for experiments at a concentration of 20% w/w. The data, together with the SLD profiles, are shown in Figures S10,S11, respectively. As before, the data are best explained by a two-layer slab model. Compared to the respective 5% w/w IL in 2-EHL systems, thinner interfacial layers and the lower SLD value of the innermost layer (particularly for [BOB]^−^) indicate an even denser interfacial ion packing and higher IL content for high-concentration systems. The [BMB]^−^ data are rather similar to those in Figure 3a, but the [BOB]^−^ data show a greater adsorption (consistent with a higher solubility) and an even larger electroresponse. The higher SLD value of the outer 2-EHL solvation layer (greater than the bulk solution SLD) is consistent with the increased D-2-EHL fraction necessary to contrast match gold at high IL concentrations. It appears that ILs are completely excluded from the first layer at the highest negative potentials for the [BOB]^−^ system, implying that the innermost cationic monolayer compensates fully the negative surface charge, and the cationic monolayer structure could interact strongly through nonpolar self-assembly with a secondary monolayer of almost pure solvent, before the bulk composition of 20% w/w IL is attained at a larger distance. This is presumably the result of the well-oriented layer with a palisade of the longer hydrocarbon chains oriented toward the bulk and able to intercalate more efficiently with the solvent.

A solvation layer is observed for each system, consistent with the shell observed in the SANS data, and even its weaker manifestation in the case of [BScB]^−^ can be inferred. Beyond that, the differences observed in SANS bear little relation to the NR observations. Clearly, the anion architecture has a profound influence on the electro-interfacial behavior. Numerous studies have observed that the structural variations in chelated orthoborate anions significantly impact surface activity, lubrication properties as well as chemical stability, and these data provide a molecular-level insight.^26,28,30,38^ The smaller [BOB]^−^ anion, without the aromatic decoration, is a little less anisotropic, whereas [BMB]^−^ and [BScB]^−^ anions have the potential for additional, directional intermolecular (aromatic) interaction modes compared to the [BOB]^−^ anion,^26^ all of which lead to not only lower affinity with solvent but also lower sensitivity to the effect of electric fields. Additionally, the larger O–B–O bond angle in the [BScB]^−^ anion (cf. Figure 1), featuring two six-membered heterocycles, integrating aromatic groups (as opposed to tethered as in the case of [BMB]^−^ anion) appears to lead to a strong ion pairing and reduces the ability to form extended structures in bulk.

Recently, bis(oxalato)borate-based ILs have been employed to enhance the electrochemical stability of alkali-metal-based batteries through forming a protective solid electrolyte interphase (SEI) to reduce consumptions of electrode and electrolyte.^73−75^ The mechanism of SEI layer formation of these systems is still unclear.^76^ The distinct electro-interfacial behavior and mobility of [BOB]^−^ anion discussed earlier, especially its structural transition phenomena observed for +0.25 and −1 V, would bring molecular insights for understanding their SEI layer formation and would further render [BOB]^−^ systems relevant for energy applications, as well as in electroactive lubrication.

#### 2.5% w/w [P_6,6,6,14_][BMB] and 2.5% w/w [P_6,6,6,14_][BOB] Mixture in 2-EHL

Both [P_6,6,6,14_][BMB] and [P_6,6,6,14_][BOB] have demonstrated promising capabilities in modifying friction and serving as antiwear agents, both as neat lubricants or additives in base oils.^28,30,44,53^ However, the tribofilm formation mechanism for these two ILs is notably different,^44,53^ which is linked to their solubility as well as the structure and density of the adsorbed film. The interfacial electroresponse is also clearly contingent upon the molecular architecture of the different anions. It is thus conceivable that a mixed formulation could be interesting in an applied context to harness the different tribofilm behavior under different friction conditions. It is therefore of interest to investigate how a mixed system would behave in terms of interfacial structuring and electroresponses under competitive conditions.

**Figure 5:**
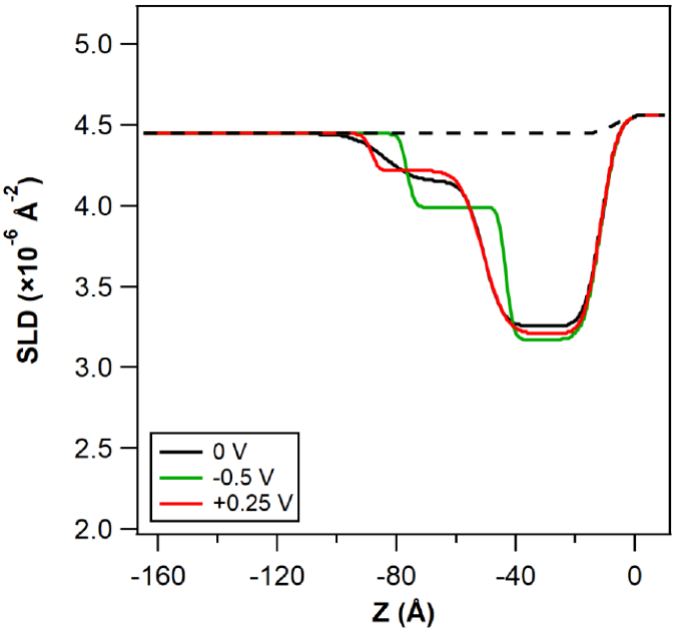
SLD profiles for the 2.5% w/w [P_6,6,6,14_][BMB] and 2.5% w/w [P_6,6,6,14_][BOB] mixture in 2-EHL solutions, obtained from the best fits to the reflectivity curves (cf. Figure S7d) as a function of distance *Z*, where the gold–solution interface is located at *Z* = 0. The potential applied order is indicated in the legend. The dashed line represents the SLD profile for the same gold surface and bulk IL/2-EHL solution that would be *predicted* in the absence of an adsorbed structured layer.

Figure 5 shows the SLD profiles for a mixture of 2.5% w/w [P_6,6,6,14_][BMB] and 2.5% w/w [P_6,6,6,14_][BOB] in 2-EHL at a gold–electrode interface at different applied potentials, derived from the best fits to the reflectivity curves (cf. Figure S7d). Detailed parameters corresponding to the SLD profiles can be found in Table S7. Additionally, the Fresnel representation for NR is provided in the Supporting Information. Note that due to differences in the molecular weights of the IL anions, the molar concentrations of 2.5% w/w [P_6,6,6,14_][BMB] and 2.5% w/w [P_6,6,6,14_][BOB] correspond to 1.1 and 1.3 mol%, respectively. The NR measurement for −1 V was not measured due to beamtime constraints.

For all potential conditions, the reflectivity curves of IL mixtures in 2-EHL were, as before, best described by a two-layer slab model (cf. FOM plot in Figure S9), aligning with the behavior of the individual IL mixtures in 2-EHL. These two-layer SLD profiles once again validate the existence of an ion-rich innermost layer and a solvent-rich outer layer, attributed to the self-assembly interaction between the [P_6,6,6,14_]^+^ cation and 2-EHL. Despite the slightly higher molar ratio of [P_6,6,6,14_][BOB] in 2-EHL, the SLD profiles of the IL mixture in 2-EHL under applied potentials show a weaker electro-responsive behavior, more closely resembling the characteristics of [P_6,6,6,14_][BMB]. Moreover, the surface charge density estimated during the equilibration is closer to that of [P_6,6,6,14_][BMB] (cf. Figure S4). Taken together, these observations suggest a higher surface affinity of the [BMB]^−^ anion.

Nonetheless, there is evidence for a hybrid nature of the film (i.e., a contribution from both [BMB]^−^ and [BOB]^−^ anions). The electroresponsivity is, while low, still a little greater than that of [BMB]^−^. Particularly at 0 and −0.5 V, the SLD is intermediate between the respective individual IL/2-EHL systems (cf. Table S7). Notably though, upon reversing the potential bias to +0.25 V, the SLD profile exhibits a reversible behavior, restoring the thickness and composition of the interfacial layer. While the slightly enhanced responsivity of the mixed IL system compared to the individual [BMB]^−^ system indicates that some [BOB]^−^ is present, this is not enough to lead to the irreversible interphase behavior seen for pure [P_6,6,6,14_][BOB] in the 2-EHL system.

The solvent-rich layer of the mixed IL/2-EHL solution is thinner under all potential conditions and also of lower SLD than for either of the individual cases. This implies a slightly enriched cation content in the outer layer (i.e., a lower solvent content). This in turn suggests more localized self-assembly interactions between the layers than in either of the pure systems, suggesting that there may be further nonadditive behavior. It remains to be seen as to whether the thinner, more cation-rich hybrid corona would have a greater load-bearing capacity than the thicker, more solvent-rich layers found in the pure systems.

Overall, [P_6,6,6,14_][BMB], with its higher interfacial preference, dominates the hybrid interfacial layers, but the presence of at least some [BOB]^−^ anions leads to a rather different orientation of the cation tails. This in turn raises the possibility that order depends on a regular EDL packing, which is broken by anions of different sizes and affinity with the cation.

## Conclusions

The bulk and electro-interfacial nanostructures of three nonhalogenated ionic liquids (ILs) in 2-ethylhexyl laurate (2-EHL) have been studied, and the influence of different anionic architectures was compared. The ILs tested contained the same quaternary trihexyl(tetradecyl)phosphonium ([P_6,6,6,14_]^+^) cation and various orthoborate-based anions (bis(mandelato)borate ([BMB]^−^), bis(oxalato)borate ([BOB]^−^), and bis(salicylato)borate ([BScB]^−^)). Small-angle neutron scattering (SANS) revealed a core–shell cylinder model with a self-assembly structure in the bulk phase of the IL/2-EHL systems. This model consists of alternating anions and the charged region of the [P_6,6,6,14_]^+^ cation as the core, surrounded by a solvent(2-EHL)-rich shell and some extended alkyl tails from the phosphonium ions, induced by self-assembly/intercalation interactions. This unexpectedly large aspect ratio structure is consistent with a linear, or 1D, self-assembly mechanism of ions or ion pairs, analogous to that earlier observed for heterogeneous nanoparticle systems.^70^ The aggregate size increases with concentration and is influenced by the anionic architecture: for [BScB]^−^, the aggregates both are shorter and have a less pronounced shell.

Neutron reflectivity (NR) confirmed the presence of a solvent-rich self-assembly structure, displaying a diffuse outer layer (solvent rich) at the gold–solution interface. The innermost layer contains a higher concentration of ionic species (e.g., IL ion pairs, cations, and anions). The composition of the near interface layer is concentration dependent, and this is most pronounced for [BOB]^−^, implying a greater solubility (and thus lower surface affinity). Under applied potentials, both interfacial layers remain distinguishable from the bulk, but the electro-responsive behavior is largely confined to the near surface layer. Distinct differences in the interfacial electro-responsive behavior were observed, reflecting the different anion properties and architectures. The [BOB]^−^ anion demonstrated the most electroresponse, followed, respectively, by [BScB]^−^ and [BMB]^−^ anions, which were nonetheless rather comparable. The [P_6,6,6,14_][BOB]/2-EHL system presented a clear electro-conditioning and ion exchange behavior with thinner, more ion-rich layers at higher absolute potentials. Conversely, the subdued electroresponse of [P_6,6,6,14_][BScB] and [P_6,6,6,14_][BMB] in 2-EHL systems can be ascribed to their lower dissociation and solubility properties, leading to a reduced ability to rearrange in response to the field.

Consequently, the [BMB]^−^ anion exhibits a higher surface affinity under competitive adsorption conditions. Importantly, the hybrid (but [BMB]^−^ dominated) films in the mixed systems actually lead to a reduced interaction with the solvent, suggesting that local disruptions of symmetry and order lead to rather different interfacial structures. This opens the way for systematic control of adsorption and electroresponse through judicious choice of ions and their ratios and will be the subject of intense future scrutiny.

These results offer insights into molecular-scale mechanisms by which electric fields modify and control the interfacial behavior of nonhalogenated IL systems and how ion architectures can influence their bulk structure. First, the application of an electric field is capable of dramatically altering the structure obtained from adsorption under neutral conditions. This altered structure, or electroactive conditioning, can then be maintained, even when the polarization is returned or even reversed. This observation has enormous implications for understanding the interfacial behavior of formulated ionic liquids in electrical applications from tribotronics to supercapacitors and potentially explains many apparently confusing anecdotal reports of slow interfacial dynamics and hysteretic values of the open circuit potential. Possibly even more importantly, from a practical deployment perspective, a specific ion–solvent self-assembly interaction between [P_6,6,6,14_]^+^ cations and 2-EHL is revealed. This is of importance for lubrication systems utilizing related nonhalogenated ILs and biodegradable ester oils, where this additional interaction will contribute to the nonsacrificial protective boundary layers, reducing both friction and wear and contributing to enhanced device life with reduced energy consumption.

## Supporting Information Available

Supporting Information for SANS and NR measurements of manuscript, additional 20% w/w [P_6,6,6,14_][BMB], and [P_6,6,6,14_][BOB] in 2-EHL NR measurements (PDF)
