# Supplementary material for: Anion Architecture Controls Structure and Electroresponsivity of Anhalogenous Ionic Liquids in a Sustainable Fluid
Source: J Phys Chem B. 2024 Apr 19;128(17):4231–42. doi: 10.1021/acs.jpcb.3c08189 (PMC11075085; doi:10.1021/acs.jpcb.3c08189)
Supplement: Supplementary file 1 — jp3c08189_si_001.pdf [file jp3c08189_si_001.pdf]

Supporting Information

Anion Architecture Controls Structure and Electroresponsivity of Anhalogenous Ionic Liquids in a Sustainable Fluid

*Sichao Li,[a] Oliver S. Hammond,[b,c] Andrew Nelson,[d] Liliana de Campo,[d] Michael Moir,[e] Carl Recsei,[e] Manishkumar R. Shimpi,[f,b] Sergei Glavatskih,[g,h,i] Georgia A. Pilkington,[*a] Anja-Verena Mudring,[*b,c,j] Mark W. Rutland[*a,h,k,l]*

a Division of Surface and Corrosion Science, School of Engineering Sciences in Chemistry, Biotechnology and Health, KTH Royal Institute of Technology, SE-100 44 Stockholm, Sweden.

b Department of Materials and Environmental Chemistry, Stockholm University, SE-114 18 Stockholm, Sweden.

c intelligent Advanced Materials, Department of Biological & Chemical Engineering and iNANO Aarhus University, Aarhus C 8000 Denmark

d Australian Centre for Neutron Scattering, ANSTO, Lucas Heights, New South Wales 2234, Australia.

e National Deuteration Facility, ANSTO, Lucas Heights, New South Wales 2234, Australia.

f Chemistry of Interfaces, Department of Civil and Environmental Engineering, Luleå University of Technology, Luleå SE-97187, Sweden

g System and Component Design, Department of Engineering Design, KTH Royal Institute of Technology, SE-100 44 Stockholm, Sweden.

h School of Chemistry, University of New South Wales, Sydney, NSW 2052, Australia.

i Department of Electromechanical, Systems and Metal Engineering, Ghent University, B-9052 Ghent, Belgium.

j Department of Physics, Umeå University, SE-901 87 Umeå, Sweden.

k Bioeconomy and Health Department Materials and Surface Design, RISE Research Institutes of Sweden, SE-114 28 Stockholm, Sweden.

l Laboratoire de Tribologie et Dynamique des Systèmes, École Centrale de Lyon, 69134 Ecully Cedex, France.

*** CORRESPONDENCE:**

Georgia A. Pilkington: georgiap@kth.se

Anja-Verena Mudring: anja-verena.mudring@bce.au.dk

Mark W. Rutland: mark@kth.se

KEYWORDS: Ionic liquid, SANS, NR, 2-EHL, sustainable tribology, supercapacitors, tribotronics, electrointerfacial response.

Table of Contents

1. Supplementary information for small-angle neutron scattering (SANS) measurement 1

2. Supplementary information for neutron reflectivity (NR) measurement 3

2.1. Infrared Spectroscopy 3

2.2. Surface charge estimation 4

2.3. Cyclic voltammetry for NR electrochemical cell after NR measurements 5

2.4. NR measurement of the gold electrode in air.. 6

2.5. NR measurement of IL in 2EHL under different applied potentials................................... 8

2.6. Fresnel representation for NR measurement of IL in 2EHL under different applied potentials 9

2.7. Figure of Merit (FOM) values.. 10

2.8. Fitted parameters from the best-fits to NR measurements 11

3. NR measurements of 20% w/w [$P_{6,6,6,14}$][BMB], and [$P_{6,6,6,14}$][BOB] in 2EHL 13

3.1. NR measurement of IL in 2EHL under different applied potentials................................. 13

3.2. Scattering length density (SLD) profiles.. 14

1. Supplementary information for small-angle neutron scattering (SANS) measurement

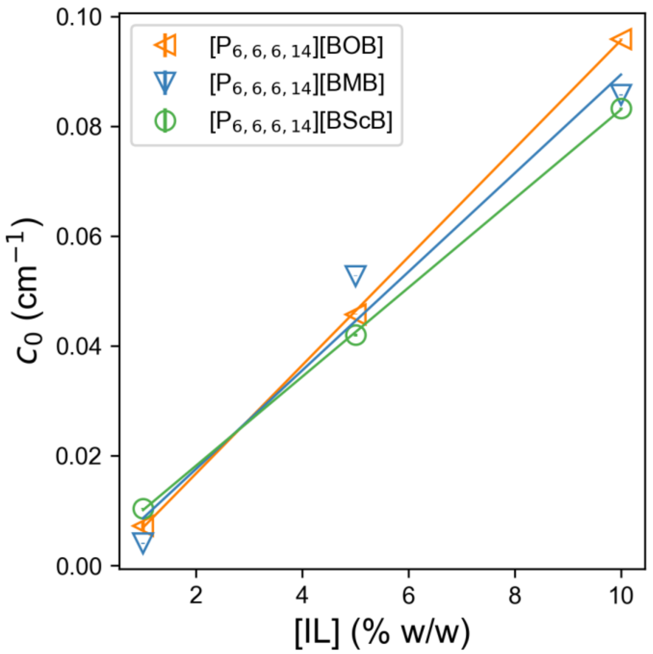

Figure S1. Fitted incoherent scattering background (c_0) as a function of IL content for the various IL in D-2EHL solutions. Linear regression fits for each IL dataset are shown as the corresponding coloured lines. The fitted values for c_0 agree well with the expected linear trend; the inelastic scattering intensity is linearly correlated with the atomic fraction of H, where the IL is the dominant source of H in these samples. This provides further confirmation that the samples are fully homogenized and match the expected composition, and that the model choice is appropriate.

Table S1. Fitted SANS scaling factors and incoherent background (c_0) for the IL in D-EHL solutions. c_0 scales linearly with the IL concentration, as shown in **Figure S1**, and the calculated goodness of fit parameter (χ^2) is included.

IL	[IL] (% w/w)	Scale	c_0 (cm^{-1})	χ^2
$[P_{6,6,6,14}]$[BOB]	1	0.081±0.067	0.007±0.001	1.2
	5	0.238±0.002	0.046±0.000	33.4
	10	0.503±0.002	0.096±0.000	144.1
$[P_{6,6,6,14}]$[BMB]	1	0.058±0.004	0.004±0.000	3.1
	5	0.325±0.002	0.053±0.000	38.4
	10	0.595±0.002	0.086±0.000	86.8
$[P_{6,6,6,14}]$[BScB]	1	0.073±0.010	0.010±0.000	1.1
	5	0.228±0.008	0.042±0.000	17.8
	10	0.399±0.003	0.083±0.000	33.1

Note: since the data are normalized to an absolute scale, we would expect that the scale factor should behave similarly, but at 1% w/w the volume fraction of scatterers is extremely small,

leading to significant uncertainties when fitted using simultaneous least-squares minimization of conjoined variables (namely scale, $\boldsymbol{\rho}_{\text{shell}}$, c_0, and the various dimensions used in the form factor).

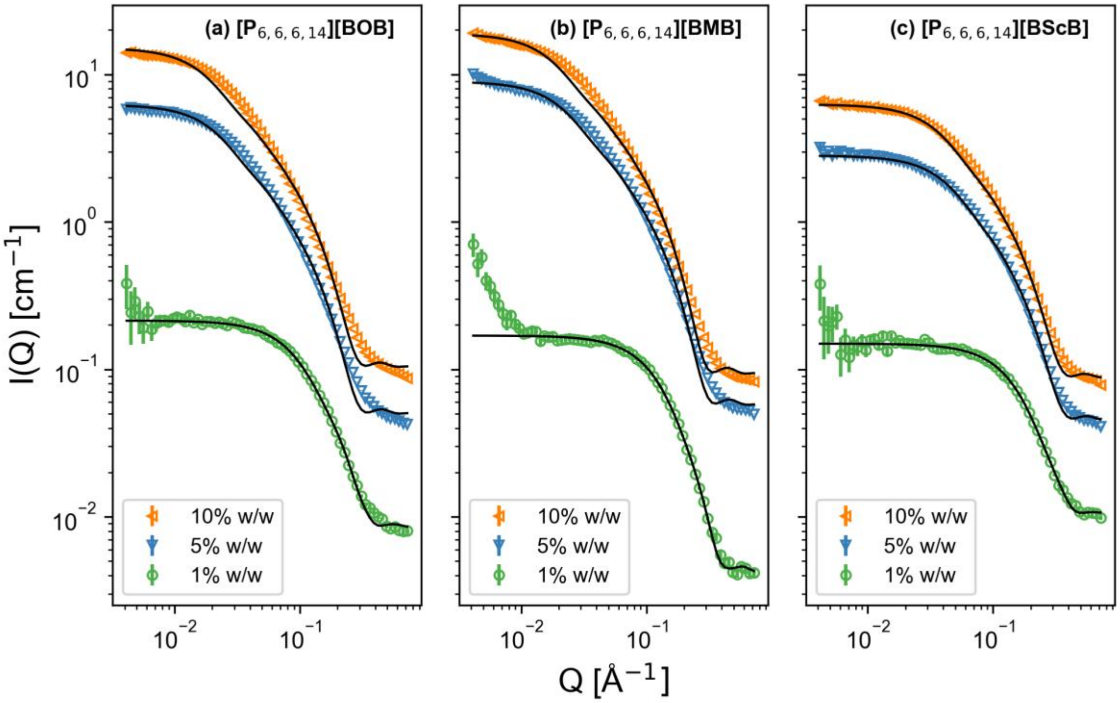

Figure S2. Fits (black lines) of SANS data (markers) using the second-best fitting model, a basic cylinder without core-shell structuring, with all fitted parameters provided in **Table S2**.

Table S2. Fitted SANS parameters for the basic cylinder (not core-shell) model, including χ^2 for comparison with the more advanced core-shell model referred to throughout the manuscript main body text. Note how the cylinder dimensions are similar, but χ^2 is significantly poorer for this basic model without a shell; χ^2 is *ca*. an order of magnitude higher, particularly for the 5 and 10% w/w samples.

IL	[IL] (% w/w)	Scale	c_0 (cm^{-1})	*L* (Å)	*R* (Å)	χ^2
[$P_{6,6,6,14}$][BOB]	1	0.004±0.000	0.008±0.000	47.8±0.5	9.0±0.1	2.9
	5	0.019±0.000	0.050±0.000	197.5±0.5	11.4±0.0	492.9
	10	0.037±0.000	0.104±0.000	239.6±0.5	11.6±0.0	1819.7
[$P_{6,6,6,14}$][BMB]	1	0.005±0.000	0.004±0.000	37.7±0.4	8.9±0.1	3.4
	5	0.031±0.000	0.057±0.000	194.2±0.5	11.1±0.0	585.8
	10	0.050±0.000	0.093±0.000	237.5±0.5	11.5±0.0	1509.0
[$P_{6,6,6,14}$][BScB]	1	0.006±0.000	0.010±0.000	30.1±0.3	7.8±0.1	1.7
	5	0.029±0.000	0.045±0.000	97.2±0.2	9.1±0.0	89.4
	10	0.051±0.000	0.088±0.000	113.2±0.2	9.4±0.0	310.4

2. Supplementary information for neutron reflectivity (NR) measurement

2.1. Infrared Spectroscopy

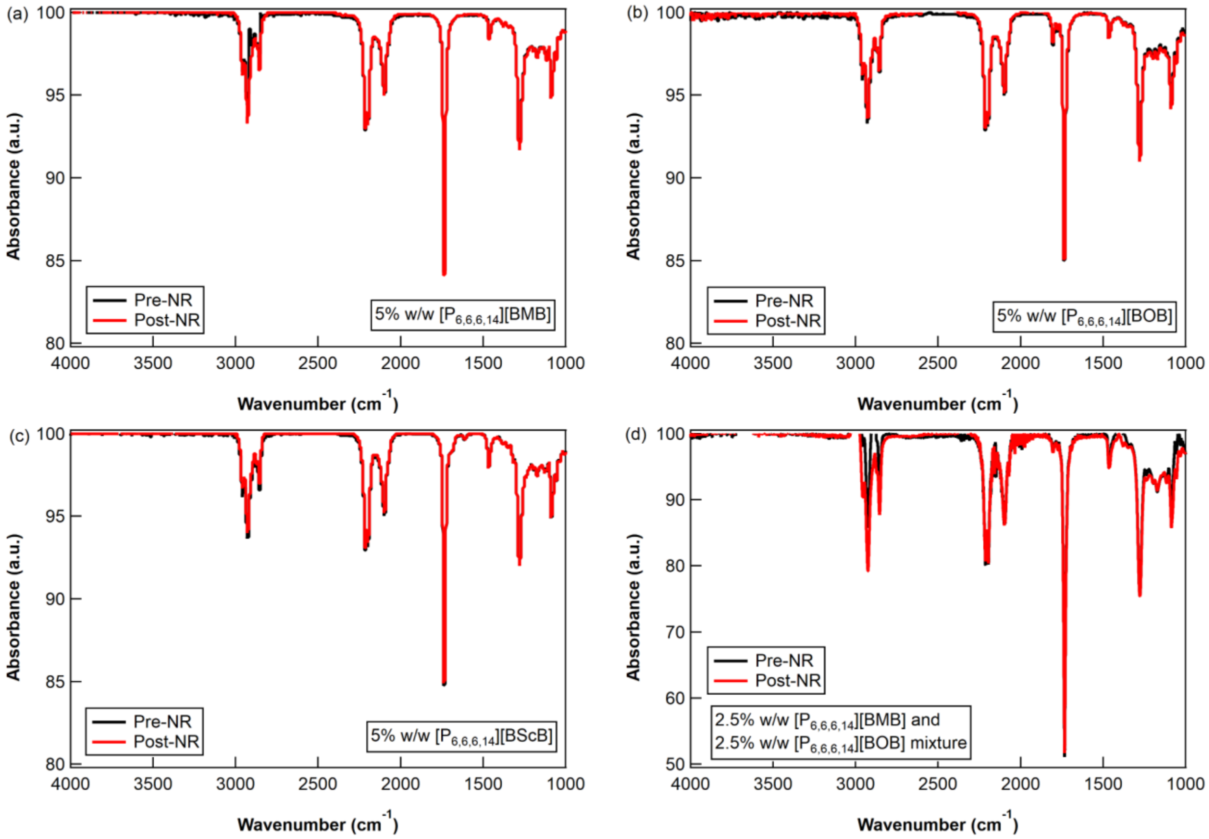

Figure S3. IR spectra over the wavelength range of 1000-4000 cm^{-1} for IL in 2-ethylhexyl laurate (2EHL) solutions before and after NR measurements**.**

2.2. Surface charge estimation

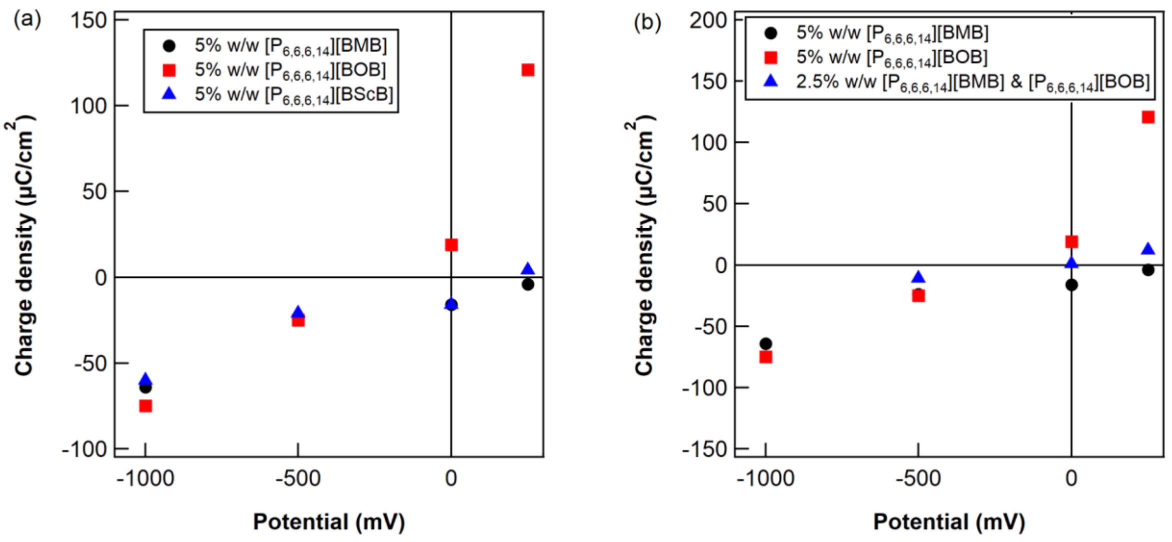

Figure S4. Surface charge density estimation after equilibrium for IL/2EHL solutions. Note that the data of 5% w/w [$P_{6,6,6,14}$][BMB] and [$P_{6,6,6,14}$][BOB] is re-plotted in **(b)** for comparison.

2.3. Cyclic voltammetry for NR electrochemical cell after NR measurements

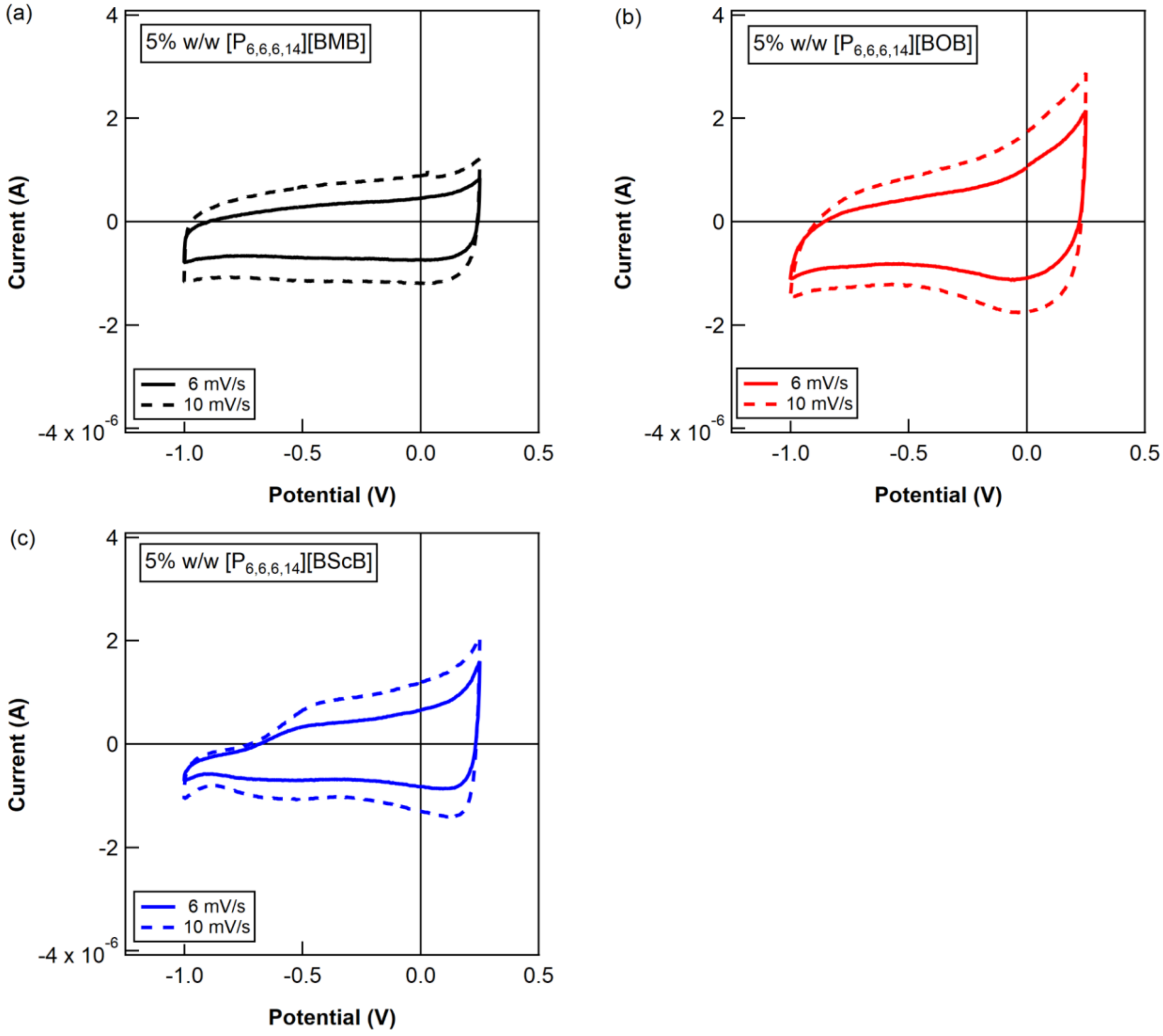

Figure S5. Cyclic voltammetry profiles for NR cell with 5% w/w [$P_{6,6,6,14}$][BxB]/2EHL (x = O, M, Sc) after NR measurements. Each system was conducted with two different scan rates: 6 mV/s and 10 mV/s. Note that CV measurement was not conducted for 2.5% w/w [$P_{6,6,6,14}$][BMB] and [$P_{6,6,6,14}$][BOB] mixture in 2EHL due to beamtime constraints.

2.4. NR measurement of the gold electrode in air

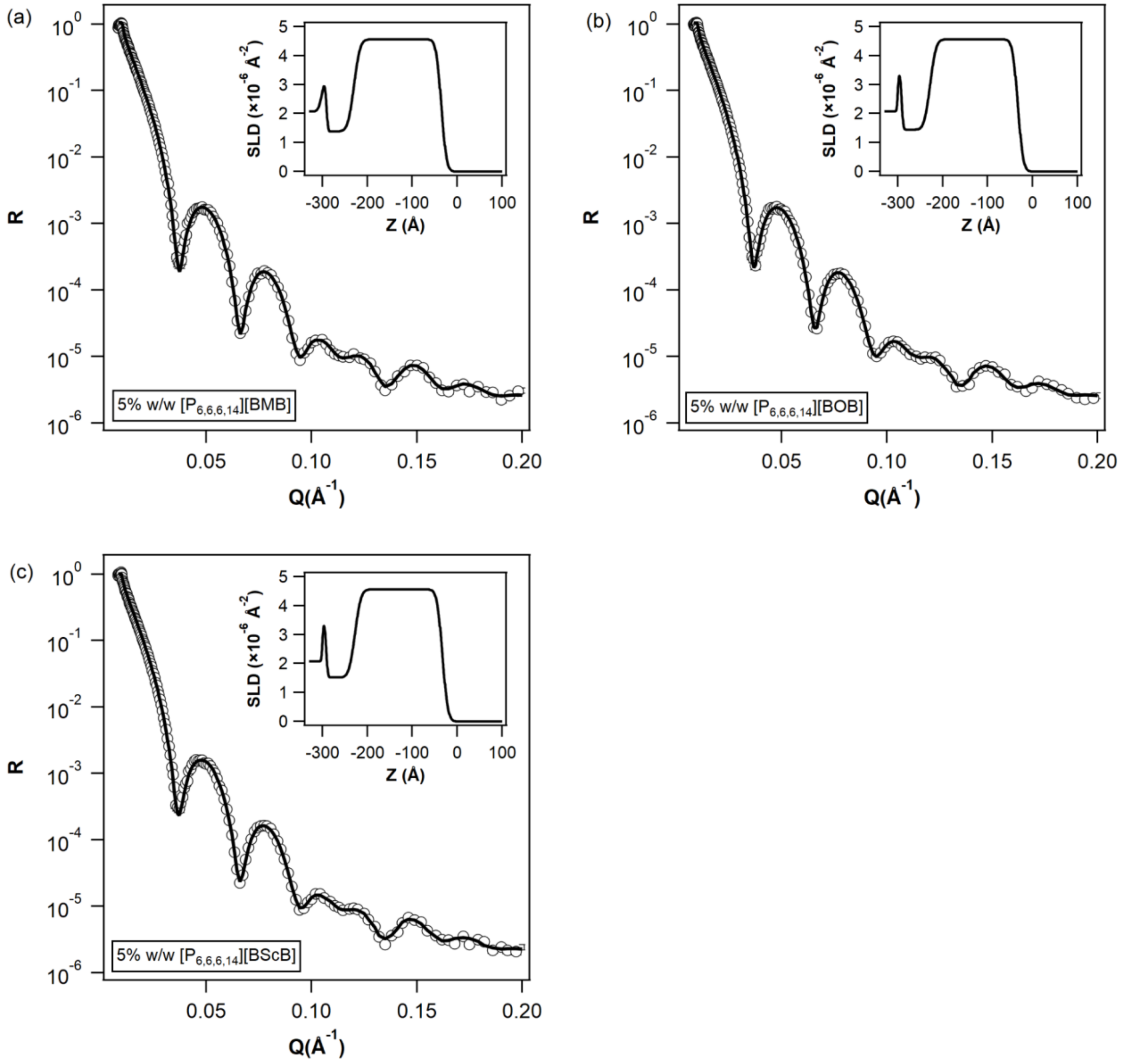

Figure S6. NR measurement of a gold electrode in air for each system. The circular points represent the experimental data, while the solid line represents a fitted scattering length density (SLD) model fit to the data. The appropriate SLD profile is displayed in the inset.

Table S3. Fitted parameters for gold electrodes in the air for each system by the model fit (solid line in the above figure).

5% w/w $[P_{6,6,6,14}]$[BMB]	Si	SiO_2	Ti	Au
Thickness (A)	∞	8.7	61.4	194.6
Roughness (Å)	7.1	3.0	10.8	9.3
SLD ($\times10^{-6}$ Å^{-2})	2.07	3.47	1.39	4.56

5% w/w $[P_{6,6,6,14}]$[BOB]	Si	SiO_2	Ti	Au
Thickness (A)	∞	8.3	63.5	195.6
Roughness (Å)	2.0	3.0	10.5	9.5
SLD ($\times10^{-6}$ Å^{-2})	2.07	3.47	1.44	4.56

5% w/w $[P_{6,6,6,14}]$[BScB]	Si	SiO_2	Ti	Au
Thickness (A)	∞	8.2	64.2	194.9
Roughness (Å)	2.0	3.0	10.7	9.7
SLD ($\times10^{-6}$ Å^{-2})	2.07	3.47	1.52	4.56

2.5. NR measurement of IL in 2EHL under different applied potentials

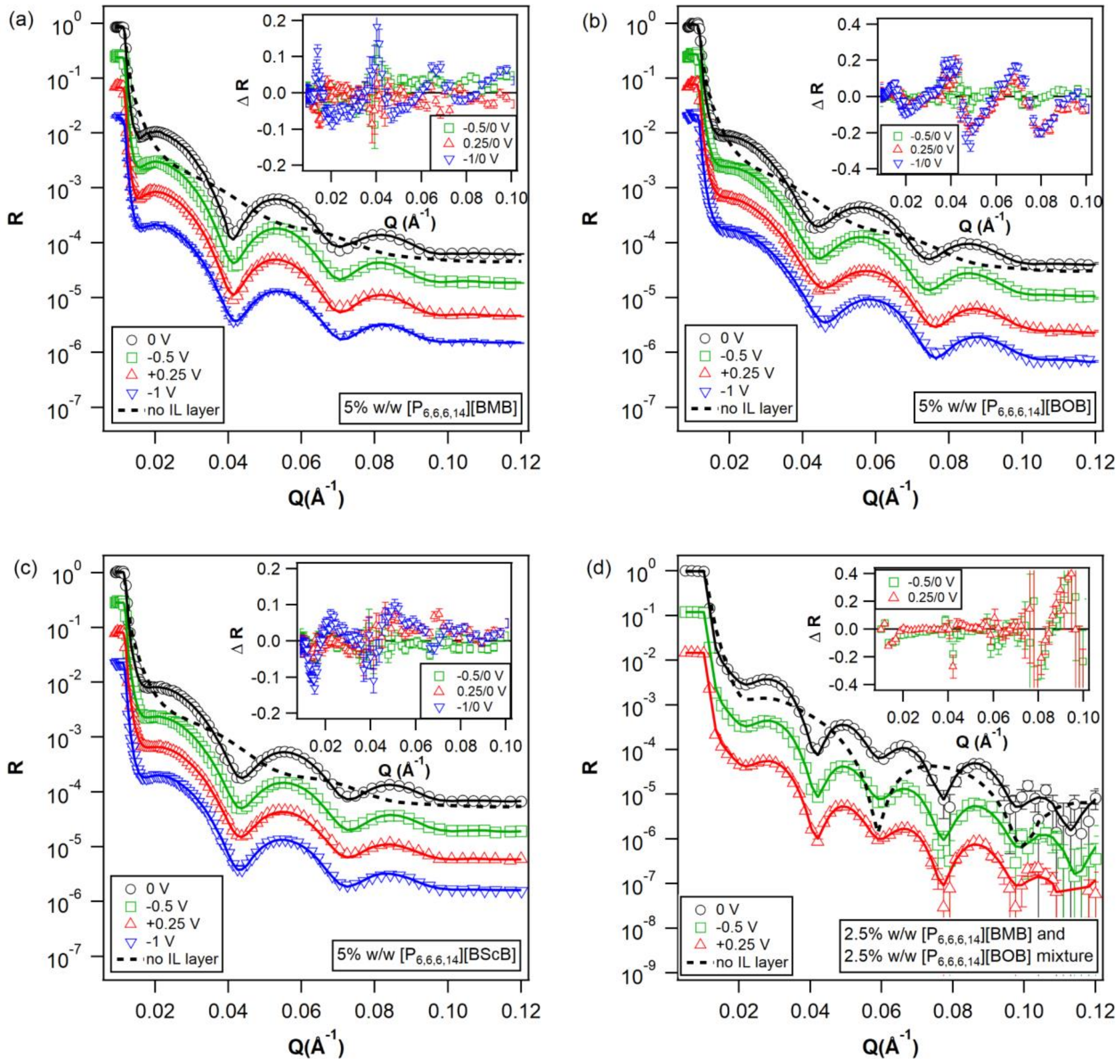

Figure S7. NR (R) of IL in 2EHL at the gold surface for different applied potentials (applied order as indicated in the legend) as a function of the momentum transfer vector Q. The symbols show the experimental data, whilst the solid lines represent the simulated NR of the best fit to the data. For clarity, the curves have been offset on the y-axis. The dashed line shows the predicted NR for the condition without interfacial IL layers. The inset shows asymmetry plots $\Delta R = \frac{[R^V(\mathrm{Q}) - R^0(\mathrm{Q})]}{[R^V(\mathrm{Q}) + R^0(\mathrm{Q})]}$, highlighting the difference of NR between polarized potentials and 0 V. Note that the data in **(b)** is reproduced from our recent study.[1]

2.6. Fresnel representation for NR measurement of IL in 2EHL under different applied potentials

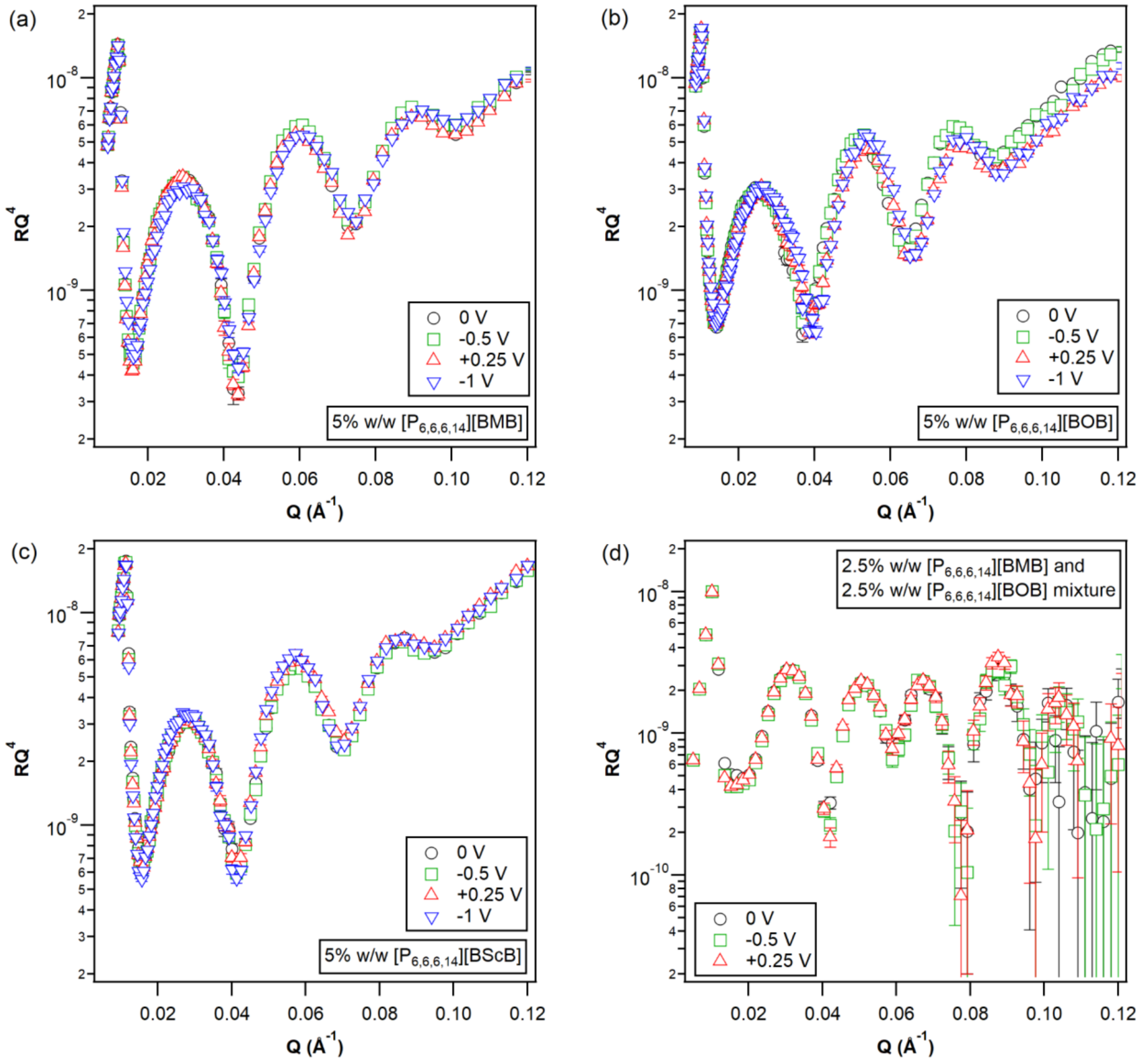

Figure S8. Fresnel representation for NR of IL in 2EHL at the gold surface for different applied potentials (applied order as indicated in the legend) as a function of the momentum transfer vector Q.

2.7. Figure of Merit (FOM) values

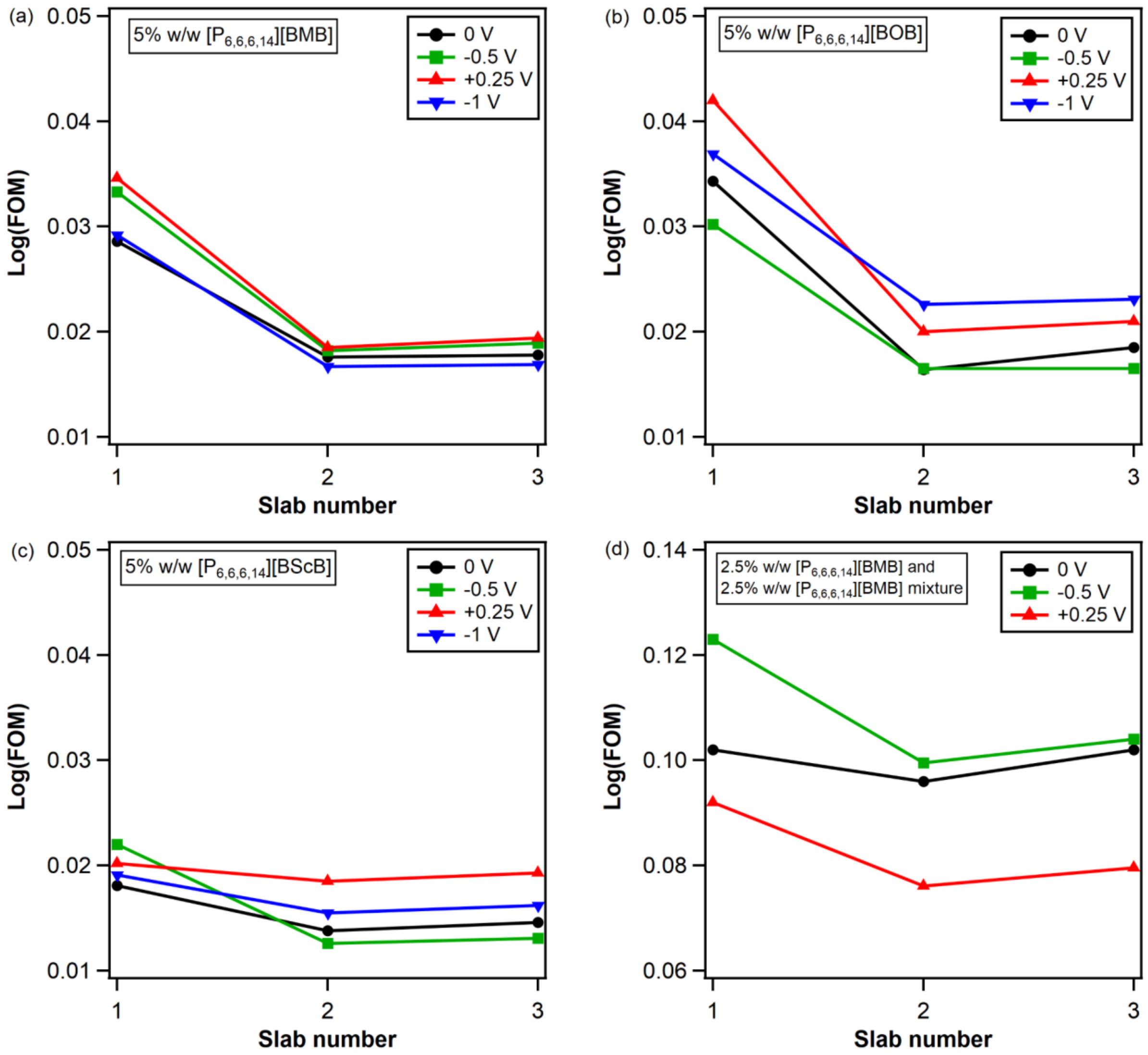

Figure S9. The FOM values for different layer slab model fitting of IL in 2EHL at the gold surface for different applied potentials. Note that different y-axis scale for 2.5% w/w [$P_{6,6,6,14}$][BMB] and [$P_{6,6,6,14}$][BOB] mixture in 2EHL **(d)** due to the different neutron reflectometer with others, SuperADAM vs. PLATYPUS.

2.8. Fitted parameters from the best-fits to NR measurements

Table S4. Fitted parameters for 5% w/w [$P_{6,6,6,14}$][BMB] in 2EHL corresponding to the model fits (solid lines) in **Figure S7a**.

Potential (V)	Layer	SLD (×10^{-6} Å^{-2})	Thickness (Å)	Roughness (Å)
0	1	3.05 [-0.05, +0.09]	20.1 [-0.9, +1.4]	6.4 [-4.2, +0.1]
	2	4.66 [-0.02, +0.02]	60.0 [-2.0, +2.8]	16.9 [-3.6, +1.2]
	Bulk	4.90 [-0.01, +0.01]	ꝏ	/
-0.5	1	3.06 [-0.04, +0.03]	21.4 [-0.6, +0.6]	3.3 [-1.3, +1.0]
	2	4.69 [-0.02, +0.02]	65.5 [-3.3, +2.1]	7.5 [-1.0, +2.6]
	Bulk	4.90	ꝏ	/
+0.25	1	2.99 [-0.05, +0.04]	20.0 [-0.7, +0.7]	6.5 [-3.5, 0]
	2	4.65 [-0.02, +0.01]	61.4 [-3.9, +1.4]	5.9 [-2.4, +4.8]
	Bulk	4.90	ꝏ	/
-1	1	3.06 [-0.03, +0.05]	17.4 [-0.5, +0.5]	3.1 [-3, 0]
	2	4.71 [-0.02, +0.01]	63.0 [-3.7, +3.7]	2.0 [-0.01, +12.8]
	Bulk	4.90	ꝏ	/

Note: Brackets show an estimation on asymmetric errors in negative and positive direction based on fitted value obtaining from Genx. These error bars are only a generic estimate of certain parameters and not quantitative values that are statistically rigorously treated.[2]

Table S5. Fitted parameters for 5% w/w [$P_{6,6,6,14}$][BOB] in 2EHL corresponding to the model fits (solid lines) in **Figure S7b**.

Potential (V)	Layer	SLD (×10^{-6} Å^{-2})	Thickness (Å)	Roughness (Å)
0	1	3.33 [-0.03, +0.03]	19.6 [-0.3, +0.4]	3.8 [-0.2, +0.6]
	2	4.72 [-0.02, +0.02]	72.2 [-4.3, +4.8]	13.6 [-1.5, +1.4]
	Bulk	4.92 [-0.01, +0.01]	ꝏ	/
-0.5	1	3.24 [-0.04, +0.03]	18.5 [-0.5, +0.2]	4.8 [-0.1, +1.0]
	2	4.77 [-0.02, +0.02]	77.1 [-2.4, +4.8]	2.0 [0, +13.4]
	Bulk	4.92	ꝏ	/
+0.25	1	2.89 [-0.06, +0.05]	9.5 [-0.7, +0.6]	3.0 [-2.0, 0]
	2	4.71 [-0.02, +0.01]	82.0 [-3.1, +4.8]	14.4 [-0.6, +3.0]
	Bulk	4.92	ꝏ	/
-1	1	2.54 [-0.08, +0.08]	7.5 [-0.9, +0.7]	2.5 [-1.4, 0]
	2	4.78 [-0.02, +0.01]	91.3 [-6.3, +5.2]	2.0 [0, +16.2]
	Bulk	4.92	ꝏ	/

Table S6. Fitted parameters for 5% w/w [$P_{6,6,6,14}$][BSB] in 2EHL corresponding to the model fits (solid lines) in **Figure S7c**.

Potential (V)	Layer	SLD (×10^{-6} Å^{-2})	Thickness (Å)	Roughness (Å)
0	1	3.32 [-0.04, +0.04]	26.0 [-1.2, +1.2]	7.3 [-1.6, +0.6]
	2	4.90 [-0.01, +0.01]	74.1 [-5.5, +1.9]	2.0 [0, +17.0]
	Bulk	4.99 [-0.01, +0.01]	∞	/
-0.5	1	3.31 [-0.02, +0.02]	25.9 [-0.4, +0.3]	5.9 [-0.5, 0]
	2	4.86 [-0.02, +0.01]	65.6 [-4.8, +1.9]	2.0 [0, +13.0]
	Bulk	4.99	∞	/
+0.25	1	3.41 [-0.05, +0.05]	28.4 [-1.3, +1.1]	7.2 [-0.6, +0.7]
	2	4.90 [-0.02, +0.02]	63.4 [-7.2, +4.1]	2.5 [0, +13.0]
	Bulk	4.99	∞	/
-1	1	3.24 [-0.02, +0.04]	29.4 [-0.4, +0.4]	5.5 [-1.0, +0.2]
	2	4.90 [-0.02, +0.01]	66.5 [-4.3, +4.6]	17.4 [-0.8, +14.9]
	Bulk	4.99	∞	/

Table S7. Fitted parameters for 2.5% w/w [$P_{6,6,6,14}$][BMB] and 2.5% w/w [$P_{6,6,6,14}$][BOB] mixture in 2EHL corresponding to the model fits (solid lines) in **Figure S7d**.

Potential (V)	Layer	SLD (×10^{-6} Å^{-2})	Thickness (Å)	Roughness (Å)
0	1	3.26 [-0.02, +0.02]	24.4 [-1.6, +1.3]	5.0 [-0.4, +0.4]
	2	4.15 [-0.05, +0.04]	33.2 [-3.9, +2.3]	8.3 [-0.8, +1.0]
	Bulk	4.45 [-0.04, +0.03]	∞	/
-0.5	1	3.17 [-0.09, +0.09]	16.5 [-5.0, +5.0]	2.0 [0, +4.0]
	2	3.99 [-0.15, +0.17]	33.2 [-4.7, +2.7]	2.0 [0, +8.0]
	Bulk	4.45	∞	/
+0.25	1	3.21 [-0.07, +0.04]	24.4 [-2.7, +1.2]	6.1 [-1.7, +1.7]
	2	4.22 [-0.10, +0.06]	37.5 [-5.2, +3.8]	2.0 [0, +8.0]
	Bulk	4.45	∞	/

3. NR measurements of 20% w/w $[P_{6,6,6,14}]$[BMB], and $[P_{6,6,6,14}]$[BOB] in 2EHL

3.1. NR measurement of IL in 2EHL under different applied potentials

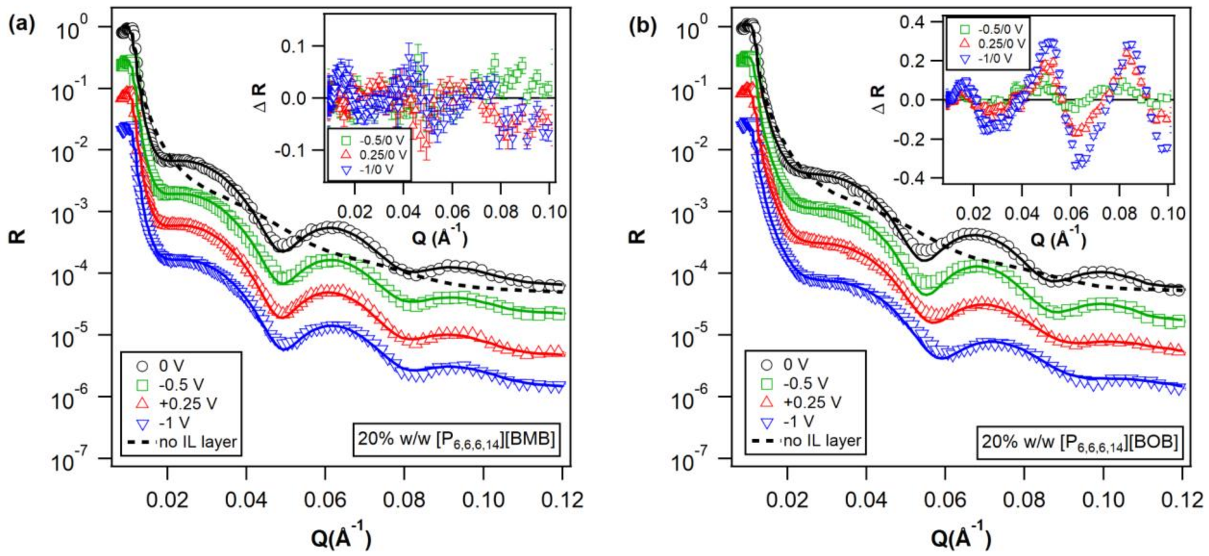

Figure S10. NR (R) of 20% w/w **(a)** $[P_{6,6,6,14}]$[BMB], and **(b)** $[P_{6,6,6,14}]$[BOB] in 2EHL at the gold surface for different applied potentials (applied order as indicated in the legend) as a function of the momentum transfer vector Q. The symbols show the experimental data, whilst the solid lines represent the simulated NR of the best fit to the data. For clarity, the curves have been offset on the y-axis. The dashed line shows the predicted NR for the condition without interfacial IL layers. The inset shows asymmetry plots $\Delta R = \frac{[R^V(\mathrm{Q})-R^0(\mathrm{Q})]}{[R^V(\mathrm{Q})+R^0(\mathrm{Q})]}$, highlighting the difference of NR between polarized potentials and 0 V.

3.2. Scattering length density (SLD) profiles

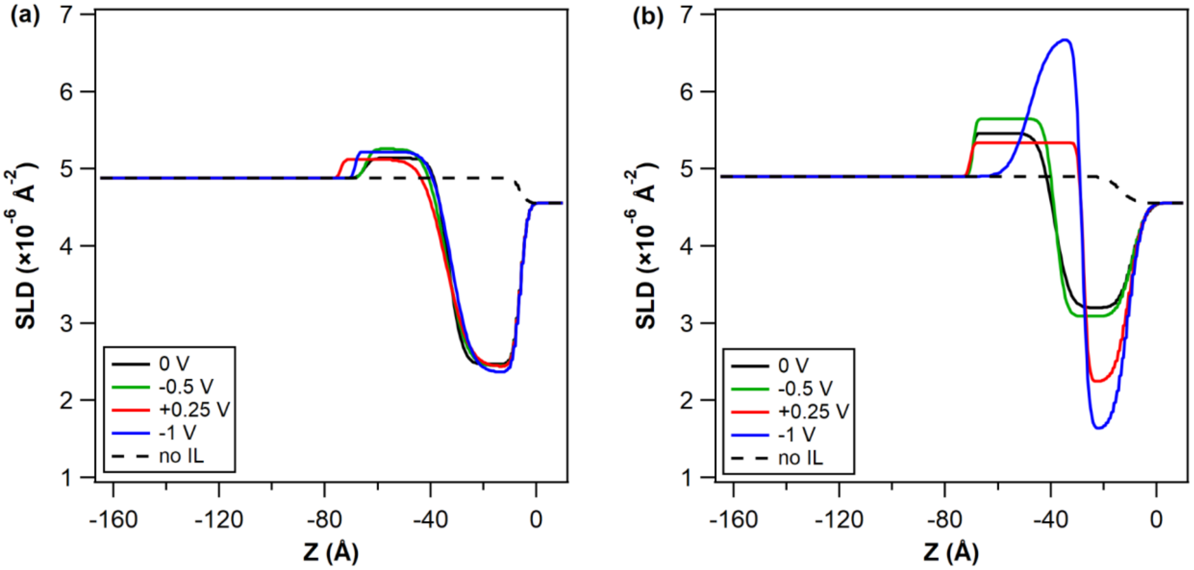

Figure S11. SLD profiles for 20% w/w **(a)** [$P_{6,6,6,14}$][BMB], and **(b)** [$P_{6,6,6,14}$][BOB] in 2EHL solutions, obtained from the best fits to the NR measurements at different applied potentials (*cf.* **Figure S10**) as a function of distance z, where the gold-solution interface is located at z = 0. The potential applied order is indicated by the legend. The dashed lines represent the SLD profile for the same gold surface and bulk IL-2EHL solution that would be *predicted* in the absence of an adsorbed, structured layer.

REFERENCES

(1) Reddy, A. B.; Pilkington, G. A.; Rutland, M. W.; Glavatskih, S. Tribotronic control of an ionic boundary layer in operando extends the limits of lubrication. *Scientific Reports* **2022**, *12* (1), 20479.

(2) Glavic, A.; Björck, M. GenX 3: the latest generation of an established tool. *Journal of applied crystallography* **2022**, *55* (4).
